# Evaluation of the detection of *GBA* missense mutations and other variants using the Oxford Nanopore MinION

**DOI:** 10.1002/mgg3.564

**Published:** 2019-01-13

**Authors:** Melissa Leija‐Salazar, Fritz J. Sedlazeck, Marco Toffoli, Stephen Mullin, Katya Mokretar, Maria Athanasopoulou, Aimee Donald, Reena Sharma, Derralynn Hughes, Anthony H.V. Schapira, Christos Proukakis

**Affiliations:** ^1^ Department of Clinical and Movement Neurosciences, Royal Free Campus, Institute of Neurology University College London London UK; ^2^ Human Genome Sequencing Center Baylor College of Medicine Houston Texas; ^3^ Institute of Translational and Stratified Medicine Plymouth University Peninsula School of Medicine Plymouth UK; ^4^ Department of Molecular Neuroscience, Institute of Neurology University College London London UK; ^5^ Department of Paediatrics Royal Manchester Children’s Hospital Manchester UK; ^6^ The Mark Holland Metabolic Unit, Salford Royal Foundation NHS Trust Salford UK; ^7^ Institute of Immunity and Transplantation Lysosomal Storage Disorders Unit, Royal Free Hospital London UK

**Keywords:** Gaucher disease, *GBA*, long‐read sequencing, mutation detection, mutation phasing, Oxford Nanopore MinION, Parkinson’s disease

## Abstract

**Background:**

Mutations in *GBA* cause Gaucher disease when biallelic and are strong risk factors for Parkinson's disease when heterozygous. *GBA* analysis is complicated by the nearby pseudogene. We aimed to design and validate a method for sequencing *GBA *using long reads.

**Methods:**

We sequenced *GBA* on the Oxford Nanopore MinION as an 8.9 kb amplicon from 102 individuals, including patients with Parkinson's and Gaucher diseases. We used NanoOK for quality metrics, NGMLR to align data (after comparing with GraphMap), Nanopolish and Sniffles to call variants, and WhatsHap for phasing.

**Results:**

We detected all known missense mutations in these samples, including the common p.N409S (N370S) and p.L483P (L444P) in multiple samples, and nine rarer ones, as well as a splicing and a truncating mutation, and intronic SNPs. We demonstrated the ability to phase mutations, confirm compound heterozygosity, and assign haplotypes. We also detected two known risk variants in some Parkinson's patients. Rare false positives were easily identified and filtered, with the Nanopolish quality score adjusted for the number of reads a very robust discriminator. In two individuals carrying a recombinant allele, we were able to detect and fully define it in one carrier, where it included a 55‐base pair deletion, but not in another one, suggesting a limitation of the PCR enrichment method. Missense mutations were detected at the correct zygosity, except for the case where the RecNciI one was missed.

**Conclusion:**

The Oxford Nanopore MinION can detect missense mutations and an exonic deletion in this difficult gene, with the added advantages of phasing and intronic analysis. It can be used as an efficient research tool, but additional work is required to exclude all recombinants.

## 1 INTRODUCTION

The *GBA* gene (OMIM #606463) encodes the lysosomal enzyme Glucocerebrosidase, deficiency of which leads to accumulation of glucosylceramide. Biallelic (homozygous or compound heterozygous) mutations in *GBA* cause Gaucher disease (GD), the most common lysosomal storage disorder (Schapira, Chiasserini, Beccari, & Parnetti, 2016). Heterozygous *GBA* mutations are a significant risk factor for Parkinson's disease (PD; Mullin & Schapira, 2015; Sidransky et al., 2009), with evidence of longitudinal changes in many carriers suggestive of prodromal PD (Beavan et al., 2015). *GBA* mutations are also associated with dementia with Lewy bodies (Geiger et al., 2016) and multiple system atrophy (MSA; Mitsui et al., 2015), related conditions which also demonstrate aggregation of the alpha‐synuclein protein. At present, more than 300 mutations have been linked to Gaucher disease (Hruska, LaMarca, Scott, & Sidransky, 2008), and the number of studies analyzing the prevalence and phenotype of *GBA* mutations in PD is rapidly increasing (Adler et al., 2017; Alcalay et al., 2015; Berge‐Seidl et al., 2017; Liu et al., 2016).

*GBA* comprises eleven exons and ten introns over ~8 kb on chromosome 1q21. A nearby pseudogene *GBAP* has 96% exonic sequence homology to the *GBA* coding region. The region also contains the Metaxin gene (*MTX1*) and its pseudogene. The existence of these two pseudogenes confers an increased risk for recombination between homologous regions, which can generate complex alleles. The homology between *GBA* and *GBAP* is highest between exons 8 and 11, where most of the pathogenic mutations have been reported, usually resulting from recombination events (Hruska et al., 2008).

The complex regional genomic structure complicates PCR and DNA sequencing, and some exons are also problematic in exome sequencing (Mandelker et al., 2016) and whole genome sequencing (Bodian et al., 2016). Established analysis protocols usually involve PCR of up to three fragments, carefully designed to not amplify *GBAP* (Neumann et al., 2009), followed by Sanger sequencing of coding exons. Illumina targeted sequencing protocols have also recently been developed (Liu et al., 2016; Zampieri, Cattarossi, Bembi, & Dardis, 2017). In recent years, long reads produced by sequencing DNA molecules in real time have become commercially available and have several advantages over short reads (Goodwin, McPherson, & McCombie, 2016). Oxford Nanopore sequencing technology analyses a single DNA molecule while it passes through a pore, producing characteristic changes in current depending on the sequence (Ip et al., 2015). The Oxford Nanopore MinION is currently the most portable long‐read sequencer. It can be plugged into a computer through a USB connection and provides sequencing data and runs metrics data in real time. It has been used for applications ranging from pathogen sequencing in the field (Quick et al., 2016) to sequencing a whole human genome (Jain et al., 2018). It is still not routinely used in human disease diagnostics, but has been successfully used for SNV detection *in CYP2D6*, *HLA‐A,* and *HLA‐B* (Sović et al., 2016); *TP53* in cancer (Crescenzio Francesco Minervini et al., 2016); and *BCR‐ABL1* in leukemia (Crescenzio F. Minervini et al., 2017). SNPs were successfully typed in chromosome 20 in a recent whole genome sequencing study of the NA12878 genome (Jain et al., 2018).

In the present study, we present and validate an efficient laboratory and bioinformatic protocol for *GBA *analysis using the MinION. In addition to disease‐causing variants, it can detect intronic ones and provide phasing information. The MinION protocol can thus provide further insights into *GBA* than other sequencing technologies and is ready to be considered for diagnostic use.

## 2 MATERIALS AND METHODS

### 2.1 Overview, DNA extraction, and PCR

Samples successfully used in this study were derived from 102 individuals. All samples shown to carry mutations are shown in Supporting Information Table S1. We used samples from saliva of 93 living individuals, and from brain from nine (seven PD and one MSA patients, and one control). Brain samples were provided by Queen Square and Parkinson's UK brain banks. SNV analyses were performed blinded to disease status and any previous sequencing results from the patient or relatives. All individuals had given written informed consent. Ethics approval was provided by the National Research Ethics Service London—Hampstead Ethics Committee, with additional permission for study of brains from the research tissue banks provided by the UK National Research Ethics Service (07/MRE09/72). DNA was isolated from brain using phenol–chloroform (Nacheva et al., 2017) and from saliva using Oragene DNA Kit.

We enriched for *GBA* by amplifying an 8.9‐kb sequence, which covered all coding exons, the introns between them, and part of the 3’ UTR region (chr1: 155,202,296–155,211,206; Supporting Information Figure S1). We customized previously reported primers (Jeong et al., 2011) to carry Oxford Nanopore adapters and barcodes for multiplexing. Primer sequences were npGBA‐F: 5’‐TTTCTGTTGGTGCTGATATTGCTCCTAAAGTTGTCACCCATACATG‐3’ and npcMTX1: 5’‐ ACTTGCCTGTCGCTCTATCTTCCCAACCTTTCTTCCTTCTTCTCAA‐3’.

Two DNA polymerases with appropriate optimized PCR conditions were used to amplify the *GBA* target region (Supporting Information Table S2): Expand Long Template PCR (Roche) and Kapa Hi‐Fi Polymerase (Kapa Biosystems). Amplicons were purified by Qiaquick PCR Purification Kit (Qiagen), and DNA concentration was measured by Qubit.

### 2.2 Barcoding, library preparation, and sequencing

For sample multiplexing, a barcoding step was carried out after generating the *GBA* amplicons with PCR Barcoding Expansion Kit 1 (up to 12 samples) or 96 (up to 96 samples) (Oxford Nanopore). We used the manufacturer amplicon sequencing protocol, starting with 1 µg of DNA and 1% ƛDNA CS spike‐in for the dA‐tailing step, followed by purification using AMPure beads. Nanopore adapters were ligated to the end‐prepped DNA, using the NEB blunt/TA ligase master mix recommended by the manufacturer. Flow cell priming was performed according to the requirements of each flow cell version. We first used R7.3 and R9 flow cells with 2D reads, where a molecule passes through the pore in both directions. After recent technical advances, we used 1D reads from R9.4 flow cells.

### 2.3 Bioinformatic analysis

MinKNOW versions 0.51.1.62 and later were used for data acquisition and run monitoring. Metrichor versions v2.38.1033–v2.40.17 were used for basecalling, de‐multiplexing, and fast5 file generation. The software divides reads into “pass” and “fail,” and only “pass” reads were analyzed. We used NanoOK (version 1.25; Leggett, Heavens, Caccamo, Clark, & Davey, 2015) to obtain a wide range of quality control metrics. This was combined with GraphMap alignment (version 0.3.0; Sović et al., 2016), using the precise region targeted as reference. We first converted fast5 files to fastq using NanoOK or Poretools (version 0.6.0; Loman & Quinlan, 2014) with a 2‐kb size cutoff. NanoOK output included the N50 (the size at which reads of the same or greater length contain 50% of the bases sequenced), the commonest erroneous substitutions, and overall error estimates, notably the aligned base identity excluding indels (ABID), and identical bases per 100 aligned bases including indels (IBAB). We aligned reads to the human genome (hg19) for detailed study and variant calling using GraphMap or NGMLR (version 0.2.6; Sedlazeck et al., 2018), both specifically developed for long reads. SAMtools (version 1.3.1) was used where required to merge, sort, downsample, and index bam files. Coverage was calculated using BEDtools (version 2.25.0; Quinlan, 2014). Data were viewed on IGV (version 2.3.9).

We used Nanopolish (versions 0.6‐dev and 0.8.4; Quick et al., 2016) to call variants over our target region. Nanopolish was specially developed to improve accuracy by reanalysis of raw signals after alignment and used in a recent whole genome study (Jain et al., 2018). It relies on a hidden Markov model which calculates the probability of the MinION data at the signal level for a given proposed sequence (Loman et al., 2015). Crucially, the nanopore does not call each base individually, but emits a signal which depends on the several bases (likely six), which are traversing the pore at any given moment. Candidate SNPs are considered within the context of all possible haplotypes. For each subset of candidate SNPs, the haplotype with the largest likelihood is called the sequence for the region. Any variants in the called haplotype are assigned a quality score, which is the log likelihood ratio between the called haplotype and the reference sequence in that region. We called variants setting ploidy to 2 and invoked the “fix homopolymers” option. When using Nanopolish 0.8.4, we had to use Albacore (version 2.1.3, Oxford Nanopore) to generate fastq files for analysis. We filtered any indel calls smaller than five bases, due to the known problem of nanopore in calling these, especially in homopolymer regions (Jain et al., 2018; Sedlazeck et al., 2018). We reviewed the variant quality of all calls and visualized them on IGV. We used WhatsHap (version 0.17; Martin et al., 2016), designed to phase missense mutations in long reads and tag bam files for visualization. We used Sniffles (version 1.0.7), another tool designed specifically for such data, to call structural variants (Sedlazeck et al., 2018).

All bioinformatic commands and the bed file for exons are given in Appendix. Variant nomenclature is based on the Human Genome Variation Society guidelines (den Dunnen et al., 2016) using GenBank reference sequence NM_000157.3. The traditional numbering for *GBA* missense mutations, which omits the first 39 amino acids, is given in brackets to ensure easy comparability with previous literature. SNVs were annotated using ANNOVAR (version 2017–07–17; Wang, Li, & Hakonarson, 2010) and viewed on www.varsome.com, which provides data from dbSNP, gnomAD (Lek et al., 2016) genomes and exomes where available, and other useful metrics.

### 2.4 Sanger sequencing

Sanger sequencing was performed at Source BioScience (UK). For exons 9–11, we performed PCR to enrich for this part of the gene with primers Fragment 8–11 Forward and Reverse (Stone et al., 2000). PCR was performed on the amplified GBA gene unless otherwise specified. Primers 9 Forward and 10–11 Reverse were used for sequencing and provided good quality data from exons 9 to 11 inclusive.

### 2.5 Statistical analysis

This was performed using GraphPad Prism v.6.0 (GraphPad, CA, USA) using paired *t* test and Mann–Whitney analysis as indicated.

## 3 RESULTS

### 3.1 *GBA* missense mutation detection is possible in patients and carriers

We first performed sequencing using 2D reads on older nanopore chemistry versions R7.3 and R9. We confirmed read alignment to the gene and detected mutations in both known carriers, among nine brain samples tested (Supplementary Note 1; Supporting Information Figures S2–S4; Tables S3 and S4). With the rapid improvements in nanopore 1D chemistry and availability of R9.4 cells, we proceeded to testing more samples, mostly known to carry pathogenic mutations. We used the Kapa PCR protocol, because of a possible minimal error reduction (Supporting Information Table S4). We initially multiplexed 10 samples, eight of which were known to carry at least one mutation, including two previously tested PD brain samples carrying RecNciI and p.L483P to test reproducibility with the new chemistry. NanoOK analysis showed high base accuracy for all samples (mean 93.2%; Supporting Information Tables S3 and S5). We aligned data using both GraphMap, and the newly developed NGMLR, with a mean *GBA *coverage >300, and minimal number of reads aligning to the pseudogene (average 0.78% and 1.97% of the reads aligning to gene with GraphMap and NGMLR, respectively; Supporting Information Table S5). Reads aligning to the pseudogene were reviewed using GenomeRibbon (Nattestad, Chin, & Schatz, 2016; Supporting Information Figure S5). We noted that in alignments by NGMLR, which splits long reads into 256‐base fragments and aligns them independently, some reads appeared to be split between the gene and pseudogene. While these could in theory represent structural variants, but represented by a small number of reads, we consider chimeric molecules formed during PCR a far more likely explanation (Laver et al., 2016).

**Figure 1:**
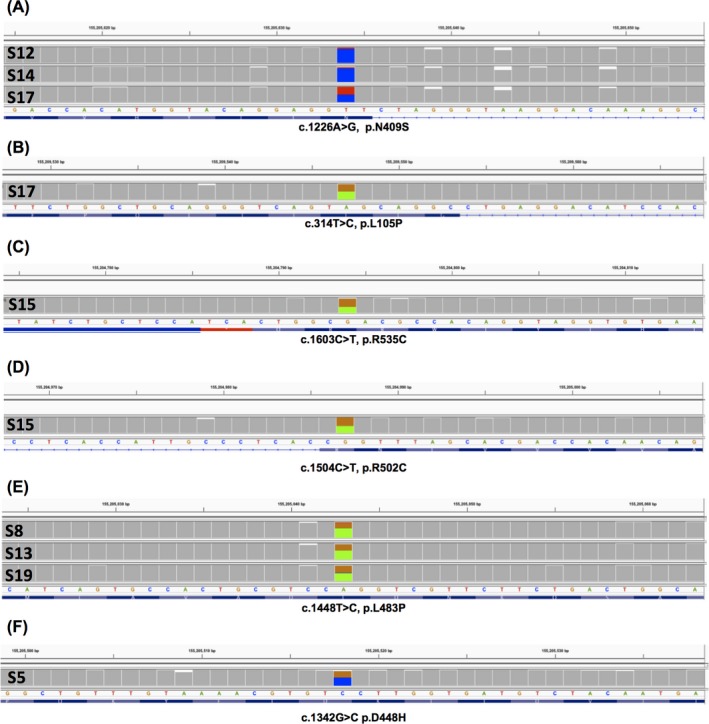
Missense mutations detected with R9.4 chemistry. The IGV trace is shown for each sample with a mutation. The mutated base is shown, with 20 bases on either side. The three SNPs which comprise RecNciI are shown in Figure 3c. GenBank reference sequence NM_000157.3

We called variants using Nanopolish (version 0.8.4) on data aligned both with GraphMap and with NGMLR. We detected all previously known coding missense mutations, at the correct zygosity, regardless of the aligner used (Table 1; Figure 1). These included p.N409S (N370S) in three GD patients, in the homozygous state in two (S12, S14), and heterozygous in one (S17) (Figure 1a), and the second mutation in S17 (p.L105P; Figure 1b). In another GD patient, we detected two other heterozygous pathogenic mutations (p.R502C, p.R535C; Figure 1c,d). In the “RecNciI” carrier (S5), in addition to the expected three coding SNVs, the p.D448H variant was reported (Figure 1f). We also detected heterozygosity in three samples from individuals without GD for p.L483P (L444P) (Figure 1e), including the one tested earlier. The Nanopolish mean quality score for coding heterozygous SNVs was 638 (standard deviation [*SD*] 229), and the lowest was 337.8. Two previously untested samples (PD patient S16 and control S18) were negative.

**Table 1:** Coding mutations detected

Genomic position	Base change	Amino acid change	Old notation	Individuals carrying	Zygosity detected
155,209,547	c.314T>C	p.L105P	L66P	1	het
155,209,430	c.431T>G	p.L144R	L105R	1	het
155,208,060	c.626G>A	p.R209P	R170P	1	het
155,207,265	c.866G>T	p.G289V	G250V	1	het
155,207,230	c.901C>G	p.R301G	R262G	1	het^a^
155,206,167	c.1093G>A	p.E365K	E326K	2	het^b^
155,206,068	c.1192C>T	p.R398^a^	R349T^a^	1	het
155,206,037	c.1223C>T	p.T408M	T369M	1	het^b^
155,205,634	c.1226A>G	p.N409S	N370S	22	hom/het
155,205,563	c.1297G>T	p.V433L	V396L	2	het
155,205,471	c.1388+1G>A	Splicing	IVS9+1C>T	2	het
155,205,542 155,205,518 155,205,043 155,205,008 155,204,994	c.[1263_1317del55; 1342G>C; 1448T>C; 1483G>C; 1497G>C]	p.L422Pfs^*^4	c.1263del+RecTL	1	het
155,205,043	c.1448T>C	p.L483P	L444P	9	het
155,205,034	c.1457T>A	p.V486E	V447E	1	het
155,204,794	c.1603C>T	p.R535C	R496C	1	het
155,204,987	c.1504C>T	p.R502C	R463C	1	het

Note

The old amino acid notation is included. The number of individuals carrying each mutation, and the zygosity in which they were detected, is shown (het = heterozygous, hom = homozygous).

GenBank reference sequence NM_000157.3

a This was initially assigned as homozygous, before RecNciI was detected (see text).

b These do not cause Gaucher disease, but are PD risk alleles.

### 3.2 Noncoding SNVs are also detected, and rare false positives can be identified

We reviewed all other SNV calls and noted several known SNPs present in the heterozygous or homozygous state, with quality scores also >500 (supporting information Table S6). We also noted seven SNVs that were reported in one or (usually) several samples with lower quality scores (all but one <200), all but one intronic (Supporting Information Table S7). These were always transitions (G>A, A>G, or C>T). These base changes were identified as common errors by NanoOK (occurring in 13.31%, 12.66%, and 11.95% on average of the relevant base, respectively). Furthermore, review of these positions on IGV in all samples revealed a high percentage of uncorrected reads with the aberrant base, including those where the SNV was not called (11%–31%; Supporting Information Figure S6). We concluded that these were false positives. Comparing the effect of the aligner on false positives, we noted that some were shared by GraphMap and NGMLR alignments from the same sample. Overall, however, the NGMLR alignments had significantly fewer false positives, mostly due to one SNP that was always called in GraphMap samples, but never in NGMLR (mean per sample 2.2 with GraphMap, and 1.2 with NGMLR; paired *t* test *p* = 0.0038).

To investigate false‐positive SNVs further, we reviewed k‐mer motifs which were prone to error according to NanoOK for the commonest false positive (chr1: 155,211,111A>G, found in four samples; no. 7 in Supporting Information Figure S6). We noted that CAGC, where the third base corresponds to the base prone to error, was within the three commonest 4‐mer errors for substitutions in all cases. Another G>A change found as a false positive in one sample (S1) was also affecting the third base in this 4‐mer. We did not notice any relation to common error in 5‐mers. We also reviewed strand bias and found an excess in false positives (*p* = 0.04; supplementary note 2). We noted several one base pair indels (deletions: mean 10, *SD* 1.41; insertions mean 0.2, *SD* 0.63) and did not analyze these any further, as small indel detection is not currently reliable.

### 3.3 Nanopolish quality score adjusted for coverage discriminates true and false positives

To investigate the effect of coverage on mutation detection, we downsampled three samples (carrying five mutations in total). The Nanopolish quality score varied linearly with coverage for true positives, but all were still reported at mean base coverage of ~50 (Supporting Information Figure S7a). They could also be discerned on IGV, which we would always recommend as a supplementary check for reported variants (Supporting Information Figure S7b). We then generated the ratio of Nanopolish quality score to coverage, to determine whether this was a more reliable discriminator than the absolute value. This was essentially constant with downsampling in a given sample (Supporting Information Figure S7c). It could distinguish true and false positives more reliably than the unadjusted quality score as there was no overlap between true and false positives, being >1.8 for true‐positive and <1.2 for false calls (Supporting Information Tables S6, S7). The mean (and standard deviation) was 3.5 (*SD* 1.7) for all true‐positive heterozygote SNVs, 2.8 (*SD* 1.1) for coding true positives, and 0.5 (*SD* 0.3) for false positives. In the two samples homozygous for p.N409S, the scores were 7.8 and 7.9, with 2.8 in a heterozygote.

### 3.4 Structural variant detection and mutation phasing provide additional relevant information

Sniffles and Nanopolish both reported a 55‐bp exonic deletion in S5 in the NGMLR alignment only, clearly visible on IGV in this alignment, and verified by Sanger sequencing (Supporting Information Figure S8). This sample had been previously designated “RecNciI” based on the presence of three pseudogene‐derived missense changes which comprise this genotype. In addition to the deletion, we detected the missense change p.D448H, both of which may coexist with the “RecNciI” mutations. This allele is classified as “c.1263del+RecTL allele,” indicating a different site of recombination with the pseudogene than RecNciI (Hruska et al., 2008). Detecting this deletion can be difficult with Illumina targeted sequencing (Zampieri et al., 2017). No other structural variants were reported.

**Figure 2:**
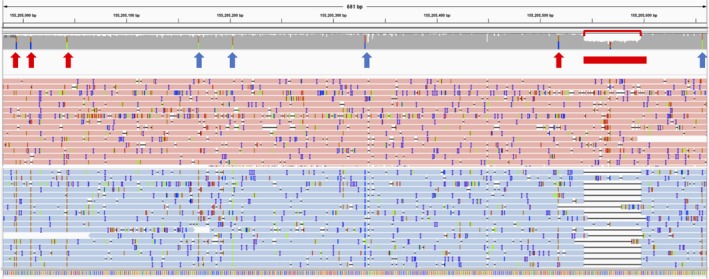
Detection and phasing of a 55‐base pair exonic deletion in S5. The coverage track, with eight SNVs highlighted, and a selection of reads are shown, over exons 9 and 10 (chr1:155,204,981–155,205,661; NGMLR alignment). The deletion is clearly visible as a drop in coverage (red bracket). Reads are grouped and colored by haplotype for these variants, which are all on the blue‐colored reads. The arrows point to the SNVs (red = coding, blue = noncoding) and the red box to the deletion. GenBank reference sequence NM_000157.3

We next phased all variants using WhatsHap (Supporting Information Table S8). We verified that the four coding SNVs and the deletion in S5 were *in cis*, as well as five rare intronic SNPs already detected in the original analysis (Figure 2). We confirmed compound heterozygosity in two GD patients, S7, heterozygous for p.N409S and p.L105P, and S15, heterozygous for p.R502C and p.R535C. We noted a haplotype comprising eight SNPs over 6.7 kb. This corresponds to the previously reported Pv1.1^+/−^ haplotype (Beutler, West, & Gelbart, 1992), later extended to a 70‐kb haplotype designated 111 (Mateu et al., 2002). One sample was homozygous and two heterozygous for Pv1.1^+^ (Supporting Information Table S8). p.N409S (N370S) was always on the Pv1.1^−^ background, as expected (Hruska et al., 2008). The p.L483P (L444P) mutation was on the Pv1.1^−^ haplotype in two individuals and the Pv1.1^+^ in one, consistent with the reported lack of founder effect (Hruska et al., 2008). p.L105P and the recombinant allele were on a Pv1.1^−^ haplotype, and p.R502C and p.R535C on Pv1.1^+^_._

### 3.5 Further multiplexing allows an efficient workflow with detection of all missense variants, but one recombinant is missed

**Figure 3:**
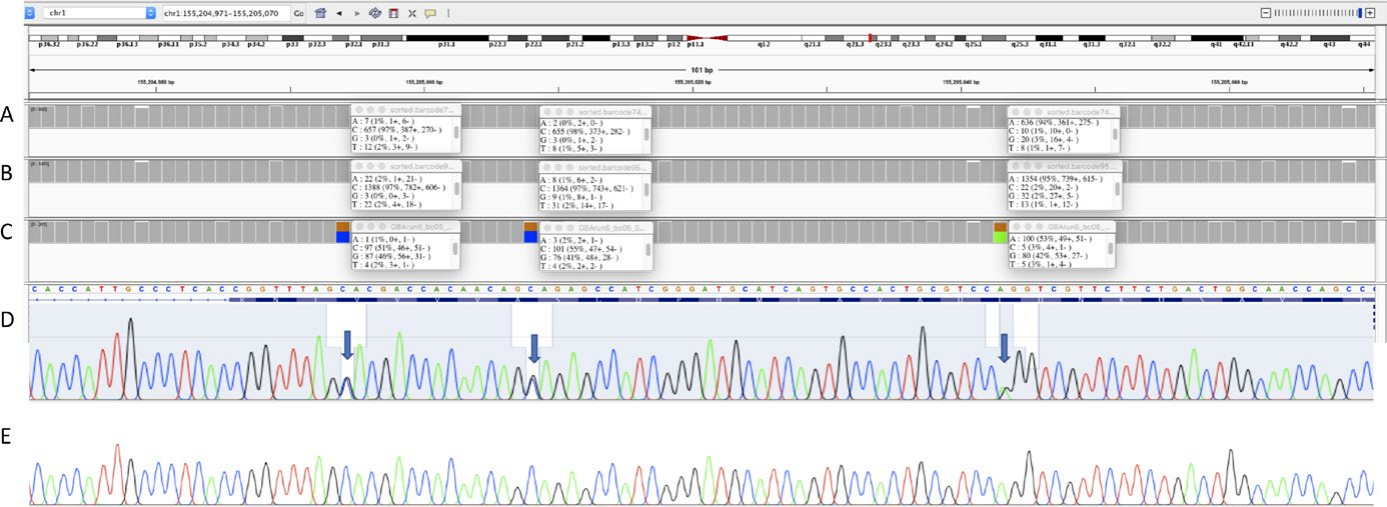
Evaluation of recombinant detection. A–C: IGV summary views over the region, including uncorrected allele frequencies at the three SNV positions. A: Nanopore sequencing does not detect even low levels of the three SNVs in bc74. B: Sample without RecNciI shown for comparison. C: The three RecNciI SNVs in this exon are clearly seen in sample S4 which carries RecTLdel55. D‐E: Sanger sequencing of exon 9–11 amplicon from bc74. D: When amplified directly from genomic DNA, RecNcil SNVs are seen (arrows). E: These are absent in nested PCR from *GBA* amplicon used for nanopore sequencing

To increase cost‐effectiveness and detect any false‐positive or false‐negative calls in a larger number, we multiplexed 92 more samples on a R9.4 flow cell. Although yields well over 5 Gb are expected, this flow cell was used after 4 months with a total data yield <4 Gb. In preparation for diagnostic use, we focused on calls in *GBA *exons and the flanking 50 bases. We considered 100 as the minimum mean coverage needed across these regions before performing analysis, which we obtained for 85 samples (mean coverage of 844, *SD* 525). These included 13 Gaucher disease patients, 11 relatives, 53 PD patients, and eight controls, four of whom were relatives of PD patients. We detected several missense mutations (Table 1) and confirmed that we had the same results as before for 12 of the Gaucher patients. One patient (bc74) appeared homozygous for p.R262G, but had been previously reported as a compound heterozygote with RecNciI (Duran et al., 2012). We performed Sanger sequencing after amplifying exons 9–11 directly from genomic DNA, which revealed the RecNciI SNVs. These were clearly absent in the nanopore sequence, and in Sanger sequencing from nested amplification of the initial amplicon, suggesting that our long‐range PCR protocol had not amplified this recombinant allele (Figure 3).

We detected one mutation in all but one GD relative, consistent with the genotype of the previously analyzed affected relative. In one sample from an obligate carrier, we did not find any mutations with the MinION, or after Sanger sequencing exons 9–11 (where we expected a mutation) after either nested or direct amplification from genomic DNA. We could not exclude sample mix‐up. Six of the PD patients (11.3%) were heterozygotes for known disease‐causing mutations, or alleles which increase the risk of PD, despite not being pathogenic for Gaucher (two p.E326K, one p.T369M; Berge‐Seidl et al., 2017). In addition, to exclude false negatives in the exons where most mutations occur, we performed Sanger sequencing of exons 9–11 for 35 negative samples. All were negative.

### 3.6 The reliability of the adjusted Nanopolish score is confirmed

We downsampled four samples, carrying six coding variants (three p.N409S, two p.L483P, and one p.E365K), by factors of 2, 4, and 10. Once again, we observed that the quality score varied linearly with coverage, but the adjusted quality score (divided by the number of reads) remained essentially constant (Supporting Information Figure S9). We reviewed all calls on IGV as before and noted an average of 1.8 false positives per sample, often repeated across samples. From seven total different false positives, five had not been seen before, but all were still transitions (Table S9). The Nanopolish quality score adjusted for the number of reads was once again a clear discriminant between true‐ and false‐positive SNVs (Supporting Information Tables S1, S9). For true‐positive heterozygotes, it was always >2.0 (mean 4.07, *SD* 1.2). For false positives, it was <1.6 (mean 0.19, *SD* 0.26) and even lower when the new false positives only were considered (mean 0.14). This suggests that the increased coverage led to additional false positives, but with low quality, and therefore easy to filter. We noted that the adjusted quality score appeared to vary with the mutation, with p.N409S significantly higher than p.L483P (mean 4.13 v 2.65; Mann–Whitney *p* = 0.001). The mean score was also higher than in the previous run, although this was partly due to the different mutations. To determine reproducibility of this score between runs, we compared the p.L483P heterozygote scores in six samples from this and three from the previous run, but the difference in this small sample size was not significant (mean 2.65 v 2.2; Mann–Whitney *p* = 0.1). We thus confirmed the ability of the adjusted Nanopolish quality score to discriminate true and false positives, and provided some evidence that this depends on the mutation, although the score may vary somewhat between experiments.

## 4 DISCUSSION

We have sequenced a long‐range *GBA* amplicon, covering all coding exons and introns, using the Oxford Nanopore Technologies MinION. We analyzed 95 samples using 1D chemistry on R9.4 flow cells, including 32 already known or expected to carry biallelic or heterozygous mutations, in a blinded fashion. We confirmed common mutations (p.N409S, p.L483P) in several samples, differentiating p.N409S homozygosity and heterozygosity. We were in total specifically able to detect the p.N409S in 4 homozygous and 19 heterozygous carriers, and p.L483P in nine heterozygotes. We also detected 12 other pathogenic mutations (nine missense, one splicing, one truncating, and one recombinant) and two missense variants which are PD risk factors. We did not detect a recombinant allele present in one sample.

Recent years have seen the introduction of single‐molecule sequencing in real time by Oxford Nanopore and PacBio which can easily generate long reads of several kb (Goodwin et al., 2016), and in the case of the nanopore up to hundreds of kb (Jain et al., 2018). Using long reads has several advantages, despite the lower accuracy at the base level (Goodwin et al., 2016), some of which were evident here. The challenge of aligning short reads to regions with high homology is often not fully appreciated (Mandelker et al., 2016), with false negatives in *GBA*‐targeted Illumina sequencing when the whole genome was used as a reference (Zampieri et al., 2017). We observed minimal alignment to the pseudogene. We also detected intronic SNPs, an understudied area in *GBA,* and other lysosomal disorders (Zampieri et al., 2017). Finally, the long reads allowed the phasing of mutations, enabling a haplotype‐resolved personalized assessment. This helps overcome the frequent problem of phasing, which may require analysis of relatives (Tewhey, Bansal, Torkamani, Topol, & Schork, 2011), as in other GBA studies (Alcalay et al., 2015).

The nanopore chemistry, and bioinformatic tools available, has evolved considerably during the time in which this work was performed. We compared two aligners (GraphMap and the more recently developed NGMLR), both of which gave negligible alignment to the pseudogene. We were able to identify and filter SNV false positives, based on (1) the low quality on Nanopolish, (2) the high % of these changes occurring as errors based on NanoOK (with some evidence of the k‐mers carrying them also being over‐represented), and (3) the significant percentage of aberrant bases at the same positions in all samples, even where not called as mutations. Notably, they were always transitions, which were also the main errors in whole genome sequencing using the MinION (Jain et al., 2018). NGMLR allowed for the detection of the 55‐bp deletion and halved the number of false positives. We thus recommend using NanoOK for quality control when testing or developing a protocol, NGMLR for alignment, Nanopolish for SNV calling, and Sniffles for structural variant calling. Nanopolish has been designed for SNV calling by correcting accuracy problems arising in nanopore default basecalling by reanalyzing the raw signal data (Jain et al., 2018). Nanopolish variant calling option uses a likelihood‐based method to generate haplotypes that serve as the reference sequence for the target region (Quick et al., 2016). It has been instrumental in projects ranging from Ebola virus (Quick et al., 2016) to human genome sequencing (Jain et al., 2018).

We noted that the ratio of the Nanopolish quality score divided by the total number of reads over that base remained almost constant with downsampling and was a very strong discriminator of true and false positives. This adjusted quality score should be useful to others analyzing this or other genes, particularly if samples with known mutations are used as controls to “benchmark” and correct for any subtle variation due to different laboratory or bioinformatic protocols. We found that coverage >100 × detected all missense and splicing variants, with no false positives after filtering. Much higher coverage slightly increased the false positives, but the additional ones had very low adjusted quality scores. Downsampling showed that lower coverage (~50×) may be adequate, but we have not validated it, particularly for zygosity determination. In a human whole genome sequencing study, coverage of only ~30x allowed SNP calling on chromosome 20 by Nanopolish with accuracy ~95%, but zygosity was not always correctly determined (Jain et al., 2018). Coverage of *GBA* was poor, so we cannot comment on SNP detection (Supporting Information Figure S10).

Current known nanopore limitations include the inability to accurately resolve homopolymers and detect small insertions and deletions (indels) (Jain et al., 2018; Sedlazeck et al., 2018), and we did not attempt to do this, filtering several single base pair indel calls. Sniffles can detect larger insertions and deletions, as demonstrated here, as well as complex structural variants (Sedlazeck et al., 2018). Based on the rapid developments in the chemistry and bioinformatics, we expect calling of small indels and further reduction of false‐positive SNV calls in the near future.

We only detected one of two *GBA* recombinant alleles. These may arise from gene conversion (nonreciprocal recombination) or gene fusion with reciprocal recombination (Tayebi et al., 2003). The latter event leads to two configurations: fusion between the gene and the pseudogene with a deletion of part of the intergenic region, and a partial duplication of the gene and pseudogene, which are fused together. In our samples, the c.1263del55+RecTL allele detected appears to be arising from gene conversion, and hence, this allele was amplified by our primers (Supporting Information Figure S11). The RecNcil allele not detected in one sample appears to have arisen as a fusion/deletion allele, with the region where our 3’ long‐range primer binds deleted. Determination of exon dosage by qPCR would help confirm this interpretation (Spataro et al., 2017). Detection of recombinants is a known problem. RecNciI can be missed by targeted Illumina sequencing, unless specifically aligning reads to *GBA*, rather than the whole genome (Zampieri et al., 2017). Recombinant alleles have been reported in PD, with frequencies of 0.7% (Liu et al., 2016) and 0.25% (Neumann et al., 2009), and may be the fifth commonest *GBA *variant in non‐Ashkenazi PD patients (Zhang et al., 2018). The frequency of all rearrangements affecting *GBA* may be even higher, as they were found in 1.6% of PD patients in one study with extensive analysis of exome data (Spataro et al., 2017). Another recent study with a combined short‐read and Sanger approach did not report any recombinants in 735 PD patients (Ruskey et al., 2018), but at least one likely recombinant, RecN370S, appears to have been missed. Other large PD studies used a variety of methods and did not report recombinant alleles, which could have been missed (Jesús et al., 2013; Kalinderi et al., 2009; Winder‐Rhodes et al., 2013). Detection of all recombinants would be possible with additional long‐range PCR using different primers, which would only yield a product if gene fusion events were present (Jeong et al., 2011). These products could then be sequenced on the MinION (or by Sanger). Ultimately, enrichment for long fragments across the entire region without PCR, for example by a CRISPR approach (Gabrieli et al., 2018), will allow comprehensive long‐read sequencing of all possible recombinants.

As treatments are now available, neonatal screening for lysosomal storage diseases is becoming commoner (Minter Baerg et al., 2017), including in some cases Gaucher (Burton et al., 2017; Hopkins et al., 2015). This relies on biochemical activity, often by blood‐spot screening (Johnson, Dajnoki, & Bodamer, 2014), with several false positives in Gaucher, possibly due to carrier status (Hopkins et al., 2015). Genetic confirmation is ultimately required, so a rapid and cost‐effective method would be useful in this setting. The advantages of the MinION include the very low capital cost, space requirements, and turnaround time of the analysis. The cost per sample is likely to compare favorably with Sanger and Illumina sequencing in all settings, especially taking into account the ability to phase variants, although Sanger validation would be sensible at least initially. Current R9.4 flow cells yields are at least 5 Gb of sequence and often much more. For our 8.9 kb amplicon, 96 samples, which is the maximum that can currently be multiplexed on a single flow cell, would therefore achieve a mean coverage >1,000×, well in excess of what is needed, even if less than a fifth of the reads aligned successfully.

Oxford Nanopore is a versatile single‐molecule real‐time sequencing technology that has been used in several innovative applications, from detection of Ebola to proof‐of‐principle human whole genome sequencing. Here, we demonstrate that the MinION can detect and phase pathogenic variants in *GBA*, and intronic SNPs that would not be detected by Sanger sequencing of exons. The rapid evolution of specific bioinformatic methods, and the improvements in accuracy and data yield, combined with the minimal footprint and capital investment, makes the MinION a suitable platform for long‐read sequencing of difficult genes such as *GBA*, both in the diagnostic and in the research environments, although additional PCR or other enrichment methods may be needed to detect a particular class of recombinants.

## CONFLICTS OF INTEREST

FJS has received honoraria and travel expenses from PacBio. CP is a participant of the Oxford Nanopore Early Access Program, was an invited speaker at the Oxford Nanopore London Calling 2018 meeting, and has received travel expenses from them.

## Appendix 1

### Summary of bioinformatic commands

#### ALBACORE to convert to fastq

read_fast5_basecaller.py ‐‐flowcell FLO‐MIN106 ‐‐kit SQK‐LSK108 ‐‐barcoding ‐‐output_format fastq ‐‐input/path/to/.fast5 ‐‐save_path/path/to/output ‐‐worker_threads 8 ‐r

#### NanoOK for QC

nanook align ‐s path/sample directory ‐r path/ref.fasta ‐aligner graphmap

nanook analyse ‐s path/sample directory ‐r path/ref.fasta ‐passonly ‐aligner graphmap

#### GRAPHMAP to align

graphmap align ‐r reference.fa ‐d reads.fasta ‐o output.sam

#### NGMLR to align

ngmlr ‐r/path/to/reference.fa ‐q/path/to/merged/.fastq ‐o/path/to/.bam

#### SAMTOOLS to sort and index bam files

samtools sort/path/to/.bam>/path/to/.sorted.bam

samtools index/path/to/.sorted.bam

#### NANOPOLISH to call variants

nanopolish index ‐d path/to/.fast5 path/to/.fastq

nanopolish variants ‐g/path/to/reference.fa ‐r/path/to/merged/.fastq ‐b/path/to/sorted.bam ‐‐ploidy 2 ‐w "1:155,202,239–155,216,653" ‐o/path/to/.vcf ‐‐snps ‐‐fix‐homopolymers

#### ANNOVAR to annotate the variants called

perl table_annovar.pl/path/to/.vcf humandb/‐buildver hg19 ‐‐protocol avsnp150 ‐‐operation f ‐nastring.‐‐outfile/path/to/annovar.vcf ‐‐polish ‐vcfinput ‐remove

#### VCFTOOLS to remove indels

vcftools ‐‐vcf/path/to/annovar.vcf ‐‐out/path/to/noindels.vcf ‐‐remove‐indels ‐‐recode ‐‐recode‐INFO‐all

#### TABIX to convert to gz and index (gzi) before using vcftools

bgzip/path/to/noindels.vcf

tabix ‐p vcf/path/to/.gz

#### VCFTOOLS to merge all.vcf files together (for easier review of output from run 1D‐2)

vcf‐merge/path/to/*.gz > merged.vcf

#### BEDTOOLS to calculate mean coverage over desired region

coverageBed ‐mean ‐a/pathto/file.bed ‐b/pathto/sorted.bam

#### BCFTOOLS to filter the merged vcf file with the bed file for exons

bcftools filter ‐R/path/to/.bed/path/to/merged.vcf.gz ‐o/path/to/filtered.vcf

#### bed file co‐ordinates used to limit calls to exons in run 1D‐2

**Table:**

1	155,210,904	155,211,119	5'UTR
1	155,210,827	155,210,903	exon1
1	155,210,371	155,210,558	exon2
1	155,209,627	155,209,918	exon3
1	155,209,357	155,209,603	exon4
1	155,208,258	155,208,491	exon5
1	155,207,875	155,208,147	exon6
1	155,207,082	155,207,419	exon7
1	155,205,986	155,206,310	exon8
1	155,205,422	155,205,685	exon9
1	155,204,936	155,205,152	exon10
1	155,204,786	155,204,941	exon11
1	155,204,189	155,204,785	3'UTR
