# Supplementary material for: Evaluation of the detection of GBA missense mutations and other variants using the Oxford Nanopore MinION
Source: Mol Genet Genomic Med. 2019 Jan 13;7(3):e564. doi: 10.1002/mgg3.564 (PMC6418358; doi:10.1002/mgg3.564)

Supplementary Figure S1. GBA gene structure. Image shows exact region targeted by our primers (chr1:155202296-155211206).

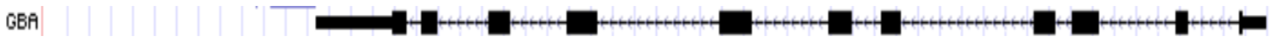

Supplementary Figure S2. Detection of known variants in S5 and S8 using 2D reads. The IGV trace over exon 10 is shown for all samples sequenced with 2D reads (see supplementary note 1).

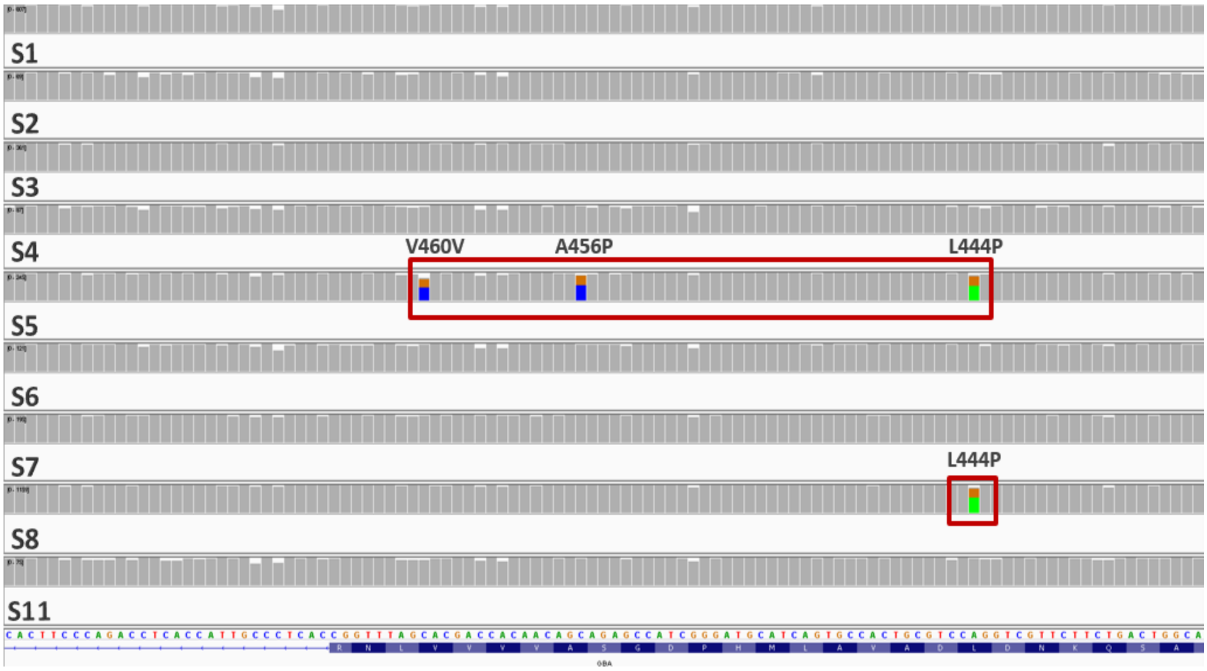

Supplementary Figure S3. Intronic variant detected in sample S1 by 2D reads. (A) IGV shows the uncorrected read count for each base at this position, consistent with heterozygosity. (B) Sanger sequencing confirmed this variant (arrow). Primer used 5’-GCGCCATCTTCACTCACTGTAAC-3’.

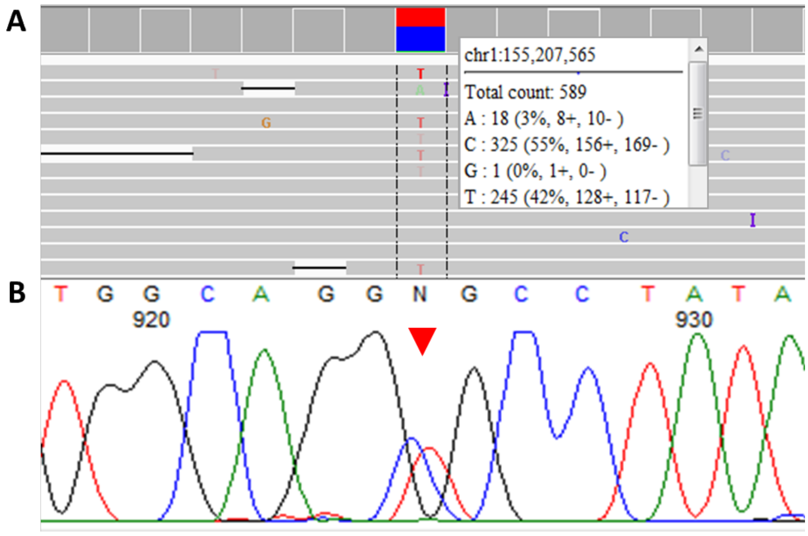

Supplementary figure S4. False positive in sample S3 (chr1: g.155210582A>G). (A) IGV coverage and uncorrected supporting reads for each base at the variant position demonstrates that it was present in 6 of the 9 samples above 10% allelic frequency, including sample S3 (red box). (B) Sanger sequencing using primer 5’-ACTGCCTTGACTCACTCACC-3’. This position (red arrow) has the reference allele. (C) NanoOK table of common substitutions shows that A>G is a common Nanopore sequencing error.

A

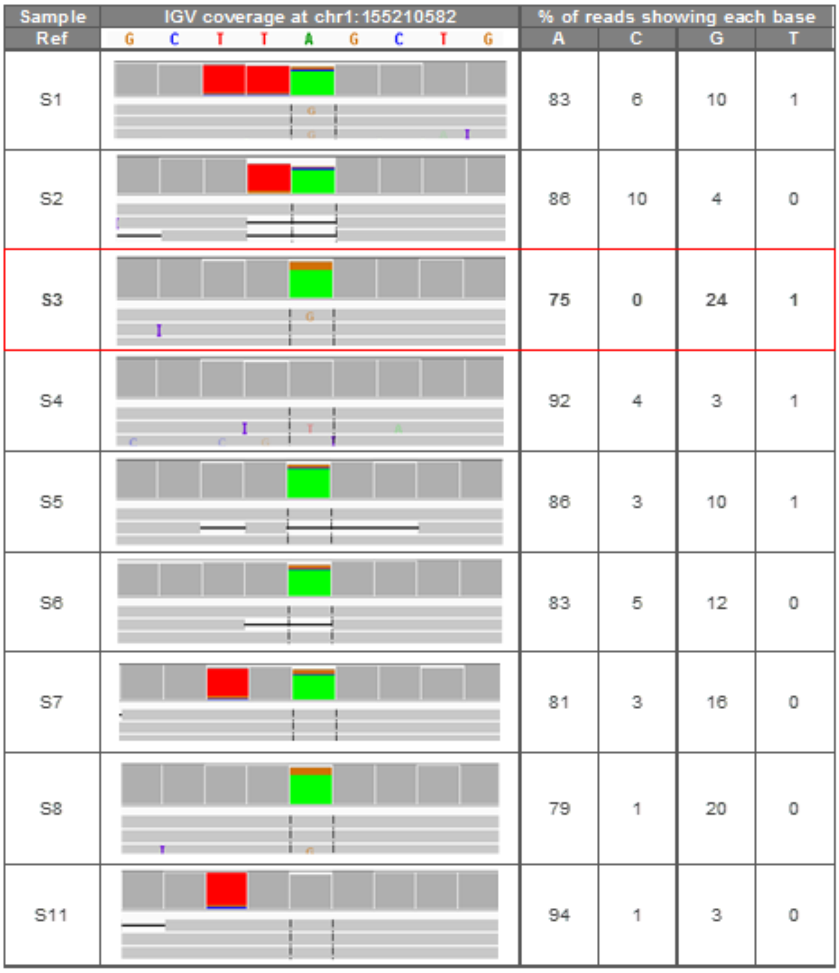

B

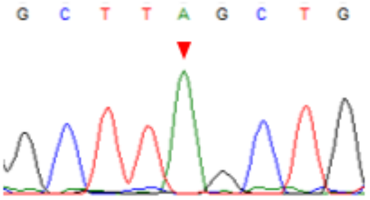

C

		2D substituted %			
		a	c	g	t
Reference	A	0.00	6.17	15.94	6.00
	C	4.31	0.00	3.94	10.56
	G	12.99	5.17	0.00	5.34
	T	5.07	19.29	5.24	0.00

Supplementary Figure S5. GenomeRibbon review of S5 alignment to gene and pseudogene.

http://genomeribbon.com (accessed 10 November 2018).

A: NGMLR alignment of all reads over gene and pseudogene, with two split reads of the correct length individually shown below. These comprise gene and pseudogene components, with two apparent transitions between gene and pseudogene in second one.

B: Graphmap alignment for same sample. Note fewer reads on pseudogene. All reads aligning to pseudogene were <5 kb, and none had split alignments. One example shown

A

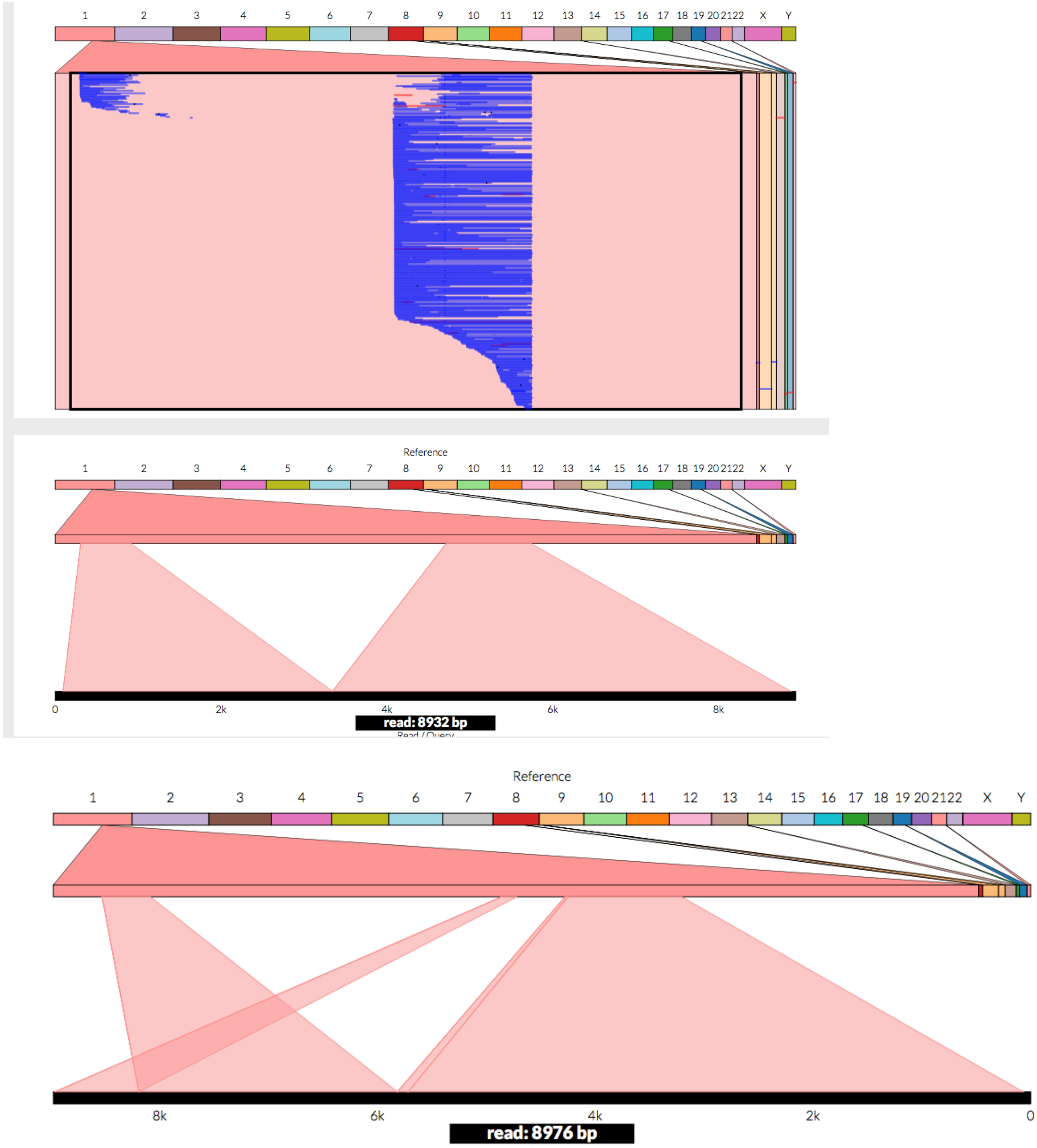

B

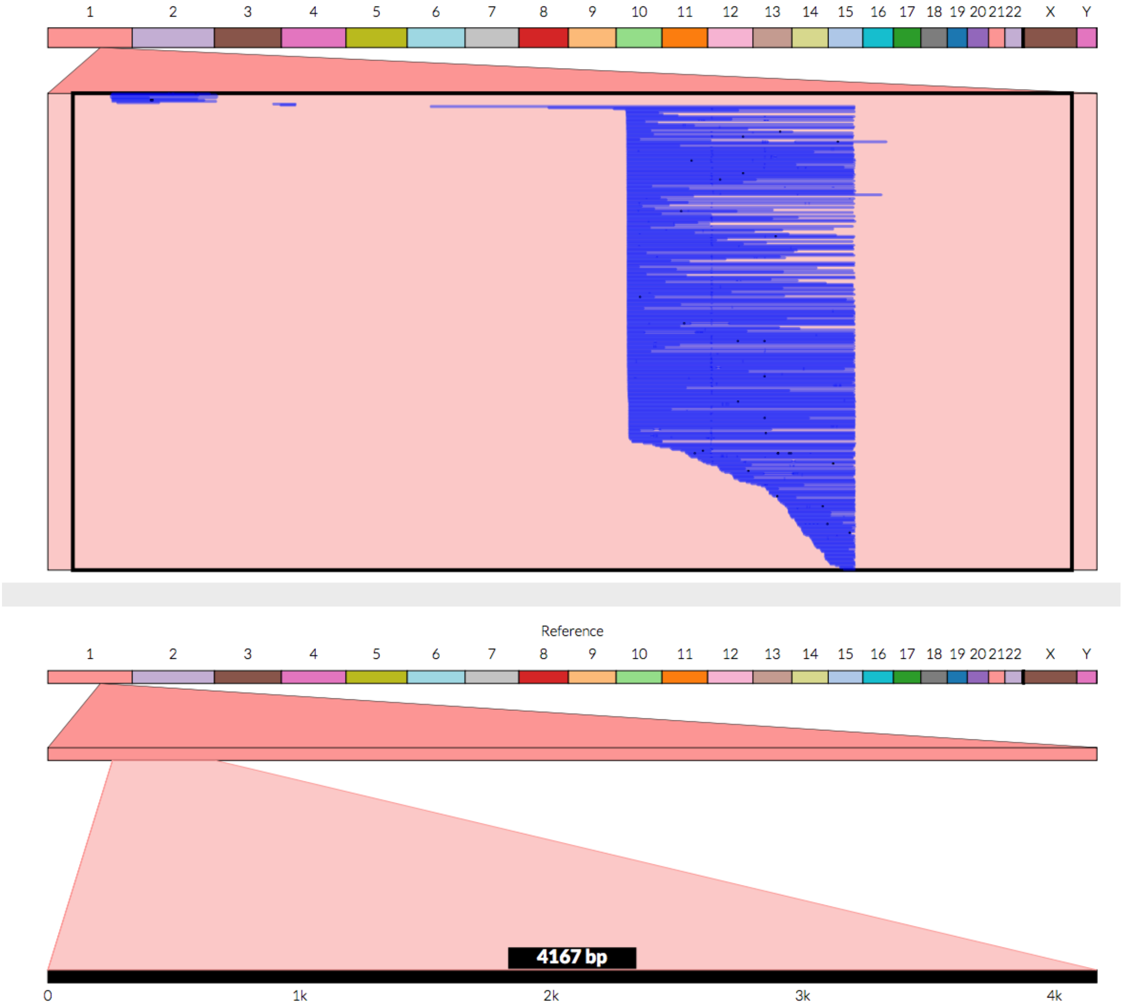
1 2 3 4 5 6 7 8 9 10 11 12 13 14 15 16 17 18 19 20 21 22 X Y
Reference
1 2 3 4 5 6 7 8 9 10 11 12 13 14 15 16 17 18 19 20 21 22 X Y
4167 bp
0
1k
2k
3k
4k

Supplementary Figure S6. False positives SNV calls with R9.4 chemistry in first run.

IGV traces (without any realignment or filtering) for all 10 samples in this flow cells are shown. The numbers correspond to Supplementary Table S8. Sample in which a given SNV was called after Graphmap alignment are denoted by ^, and after NGMLR by *. The number of uncorrected calls of each base at that position, illustrating the high number of errors at these positions in all or most samples. Note that (3) was always called in Graphmap aligned samples, and never in NGMLR-aligned ones.

(1)

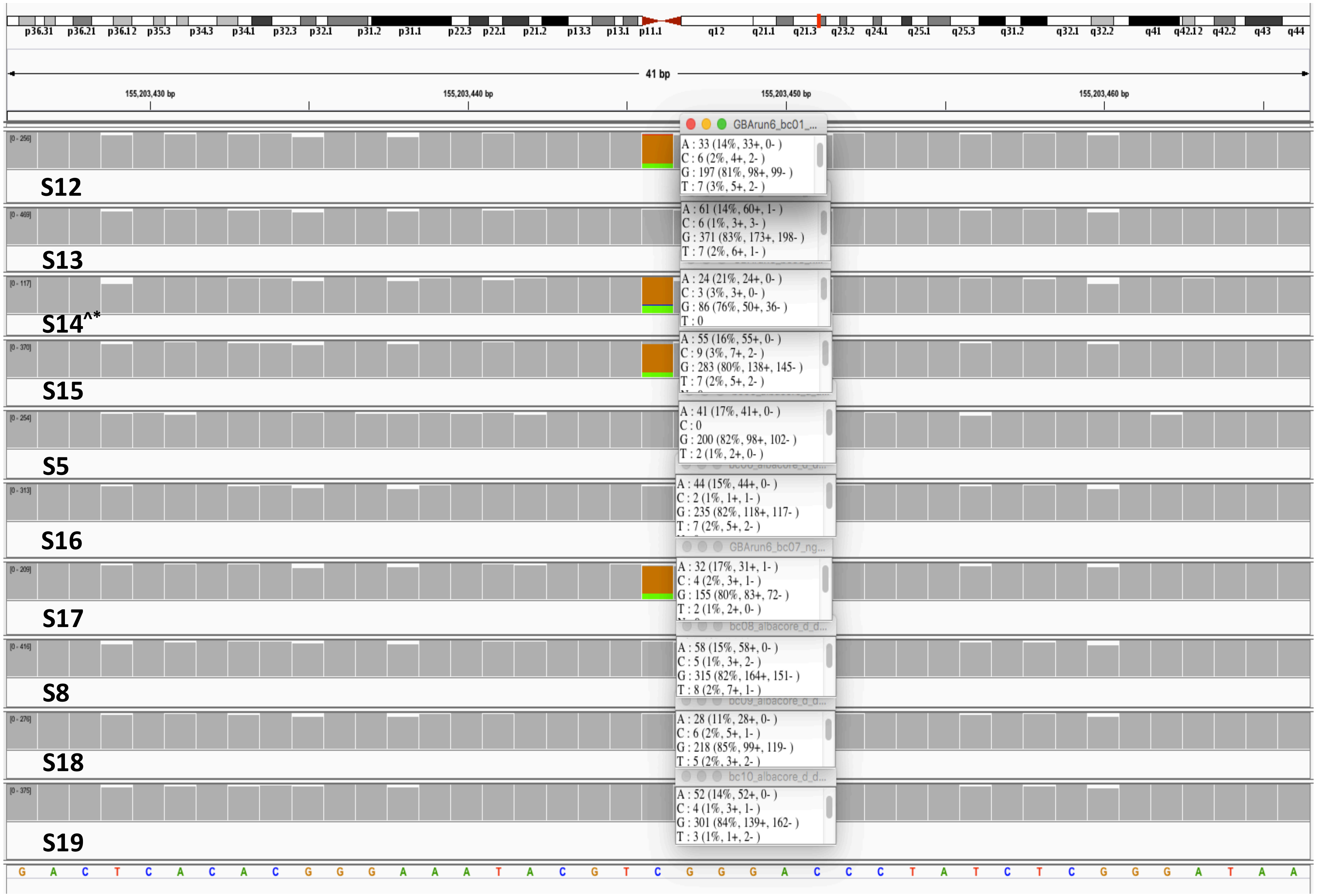
41 bp
155,203,430 bp
155,203,440 bp
155,203,450 bp
155,203,460 bp
GBArun6_bc01_...
S12
A : 33 (14%, 33+, 0-)
C : 6 (2%, 4+, 2-)
G : 197 (81%, 98+, 99-)
T : 7 (3%, 5+, 2-)
S13
A : 61 (14%, 60+, 1-)
C : 6 (1%, 3+, 3-)
G : 371 (83%, 173+, 198-)
T : 7 (2%, 6+, 1-)
S14^*
A : 24 (21%, 24+, 0-)
C : 3 (3%, 3+, 0-)
G : 86 (76%, 50+, 36-)
T : 0
S15
A : 55 (16%, 55+, 0-)
C : 9 (3%, 7+, 2-)
G : 283 (80%, 138+, 145-)
T : 7 (2%, 5+, 2-)
S5
A : 41 (17%, 41+, 0-)
C : 0
G : 200 (82%, 98+, 102-)
T : 2 (1%, 2+, 0-)
S16
A : 44 (15%, 44+, 0-)
C : 2 (1%, 1+, 1-)
G : 235 (82%, 118+, 117-)
T : 7 (2%, 5+, 2-)
GBArun6_bc07_ng...
S17
A : 32 (17%, 31+, 1-)
C : 4 (2%, 3+, 1-)
G : 155 (80%, 83+, 72-)
T : 2 (1%, 2+, 0-)
bc08_albacore_d_d...
S8
A : 58 (15%, 58+, 0-)
C : 5 (1%, 3+, 2-)
G : 315 (82%, 164+, 151-)
T : 8 (2%, 7+, 1-)
S18
A : 28 (11%, 28+, 0-)
C : 6 (2%, 5+, 1-)
G : 218 (85%, 99+, 119-)
T : 5 (2%, 3+, 2-)
bc10_albacore_d_d...
S19
A : 52 (14%, 52+, 0-)
C : 4 (1%, 3+, 1-)
G : 301 (84%, 139+, 162-)
T : 3 (1%, 1+, 2-)
G A C T C A C A C G G G A A A T A C G T C G G G A C C C T A T C T C G G G A T A A

(2)

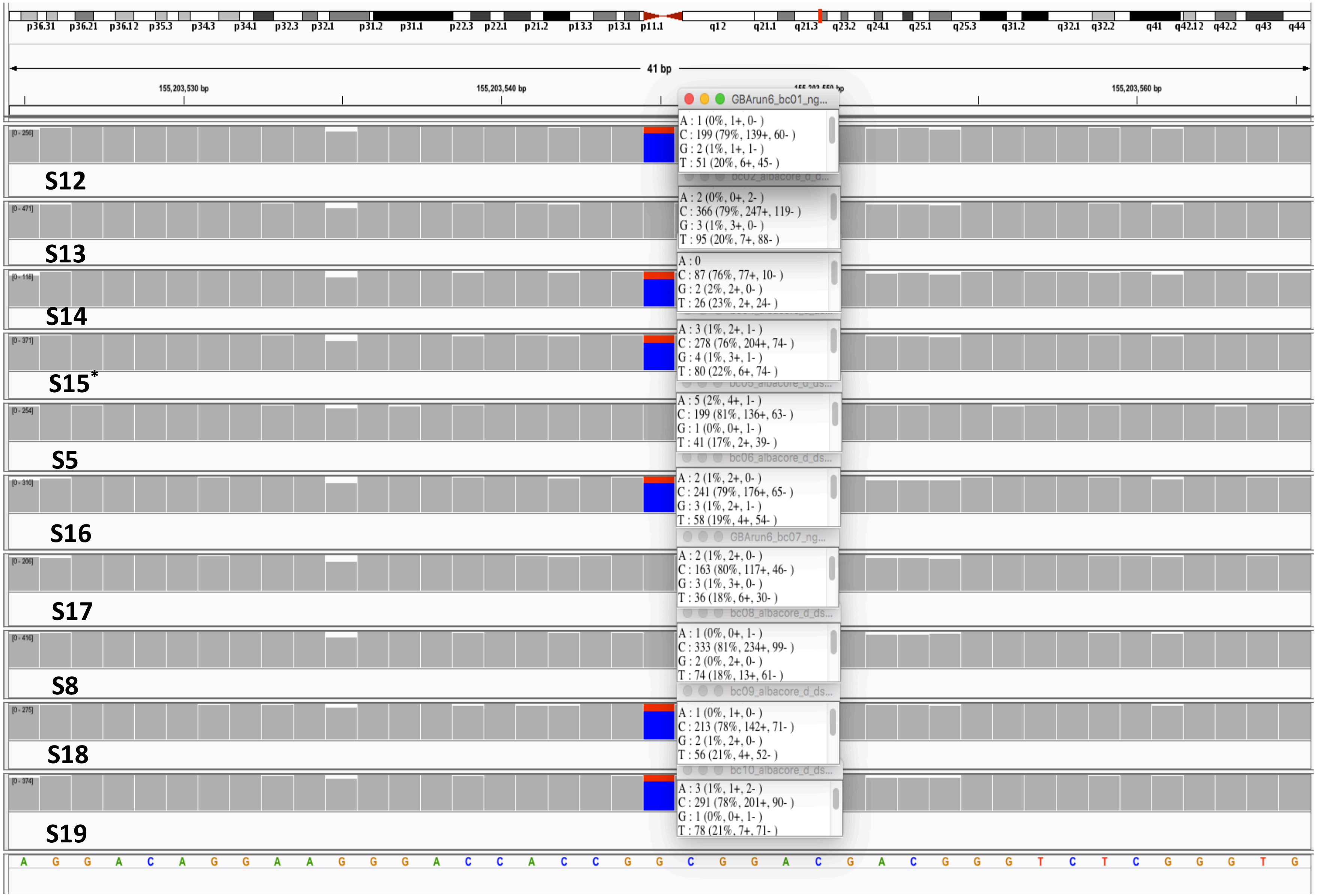
p36.31 p36.21 p36.12 p35.3 p34.3 p34.1 p32.3 p32.1 p31.2 p31.1 p22.3 p22.1 p21.2 p13.3 p13.1 p11.1 q12 q21.1 q21.3 q23.2 q24.1 q25.1 q25.3 q31.2 q32.1 q32.2 q41 q42.12 q42.2 q43 q44
41 bp
155,203,530 bp
155,203,540 bp
155,203,560 bp
GBArun6_bc01_ng...
S12
A : 1 (0%, 1+, 0-)
C : 199 (79%, 139+, 60-)
G : 2 (1%, 1+, 1-)
T : 51 (20%, 6+, 45-)
S13
A : 2 (0%, 0+, 2-)
C : 366 (79%, 247+, 119-)
G : 3 (1%, 3+, 0-)
T : 95 (20%, 7+, 88-)
S14
A : 0
C : 87 (76%, 77+, 10-)
G : 2 (2%, 2+, 0-)
T : 26 (23%, 2+, 24-)
S15*
A : 3 (1%, 2+, 1-)
C : 278 (76%, 204+, 74-)
G : 4 (1%, 3+, 1-)
T : 80 (22%, 6+, 74-)
S5
A : 5 (2%, 4+, 1-)
C : 199 (81%, 136+, 63-)
G : 1 (0%, 0+, 1-)
T : 41 (17%, 2+, 39-)
bc06_albacore_d_ds...
S16
A : 2 (1%, 2+, 0-)
C : 241 (79%, 176+, 65-)
G : 3 (1%, 2+, 1-)
T : 58 (19%, 4+, 54-)
GBArun6_bc07_ng...
S17
A : 2 (1%, 2+, 0-)
C : 163 (80%, 117+, 46-)
G : 3 (1%, 3+, 0-)
T : 36 (18%, 6+, 30-)
bc08_albacore_d_ds...
S8
A : 1 (0%, 0+, 1-)
C : 333 (81%, 234+, 99-)
G : 2 (0%, 2+, 0-)
T : 74 (18%, 13+, 61-)
bc09_albacore_d_ds...
S18
A : 1 (0%, 1+, 0-)
C : 213 (78%, 142+, 71-)
G : 2 (1%, 2+, 0-)
T : 56 (21%, 4+, 52-)
bc10_albacore_d_ds...
S19
A : 3 (1%, 1+, 2-)
C : 291 (78%, 201+, 90-)
G : 1 (0%, 0+, 1-)
T : 78 (21%, 7+, 71-)
A G G A C A G G A A G G G A C C A C C G G C G G A C G A C G G G T C T C G G G T G

(3)

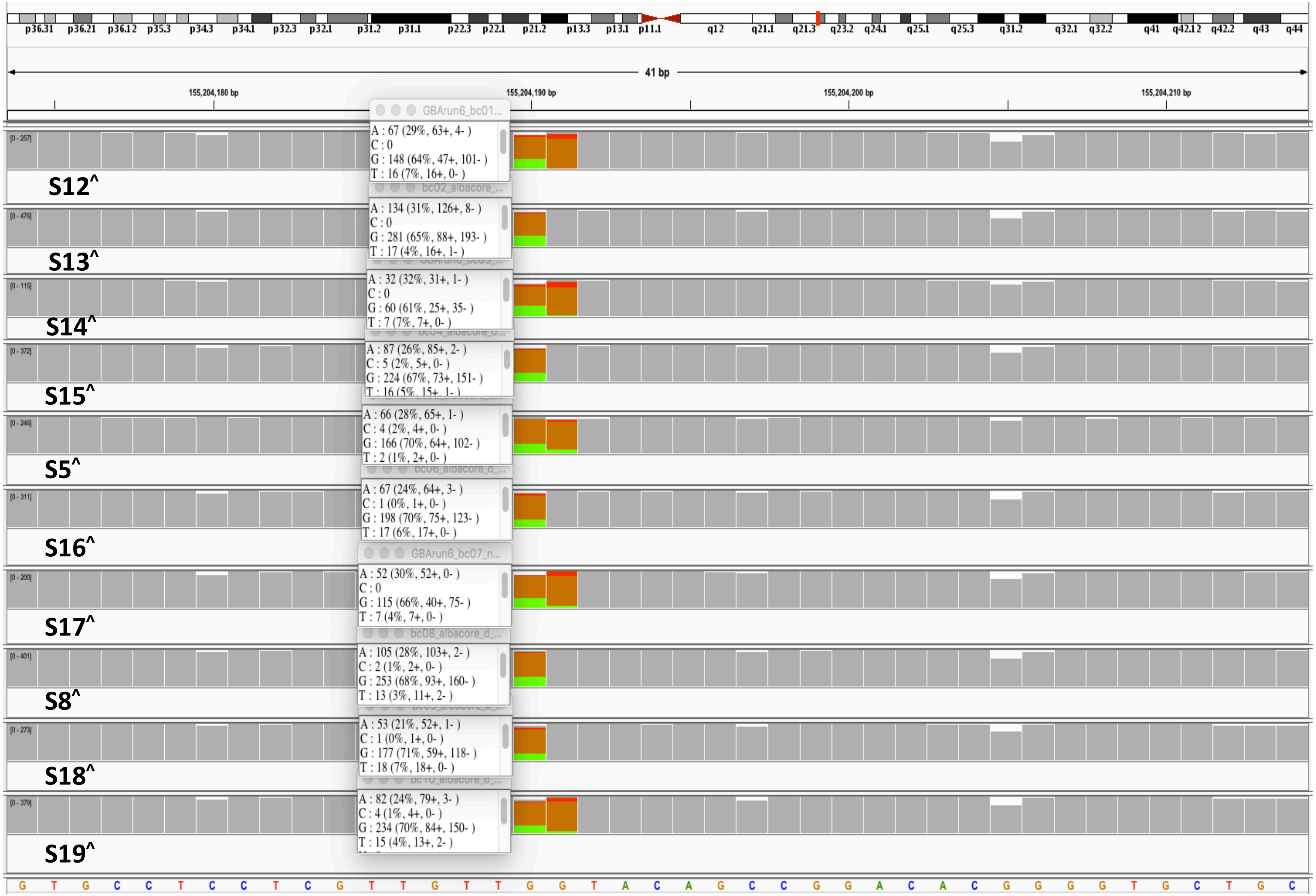
41 bp
155,204,180 bp
155,204,190 bp
155,204,200 bp
155,204,210 bp
GBArun6_bc01...
A : 67 (29%, 63+, 4-)
C : 0
G : 148 (64%, 47+, 101-)
T : 16 (7%, 16+, 0-)
S12^
bc02_albacore_...
A : 134 (31%, 126+, 8-)
C : 0
G : 281 (65%, 88+, 193-)
T : 17 (4%, 16+, 1-)
S13^
A : 32 (32%, 31+, 1-)
C : 0
G : 60 (61%, 25+, 35-)
T : 7 (7%, 7+, 0-)
S14^
A : 87 (26%, 85+, 2-)
C : 5 (2%, 5+, 0-)
G : 224 (67%, 73+, 151-)
S15^
A : 66 (28%, 65+, 1-)
C : 4 (2%, 4+, 0-)
G : 166 (70%, 64+, 102-)
T : 2 (1%, 2+, 0-)
S5^
A : 67 (24%, 64+, 3-)
C : 1 (0%, 1+, 0-)
G : 198 (70%, 75+, 123-)
T : 17 (6%, 17+, 0-)
S16^
GBArun6_bc07_n...
A : 52 (30%, 52+, 0-)
C : 0
G : 115 (66%, 40+, 75-)
T : 7 (4%, 7+, 0-)
S17^
bc08_albacore_d_...
A : 105 (28%, 103+, 2-)
C : 2 (1%, 2+, 0-)
G : 253 (68%, 93+, 160-)
T : 13 (3%, 11+, 2-)
S8^
A : 53 (21%, 52+, 1-)
C : 1 (0%, 1+, 0-)
G : 177 (71%, 59+, 118-)
T : 18 (7%, 18+, 0-)
S18^
A : 82 (24%, 79+, 3-)
C : 4 (1%, 4+, 0-)
G : 234 (70%, 84+, 150-)
T : 15 (4%, 13+, 2-)
S19^
G T G C C T C C T C G T T G T T G G T A C A G C C G G A C A C G G G G T G C T G C

(4)

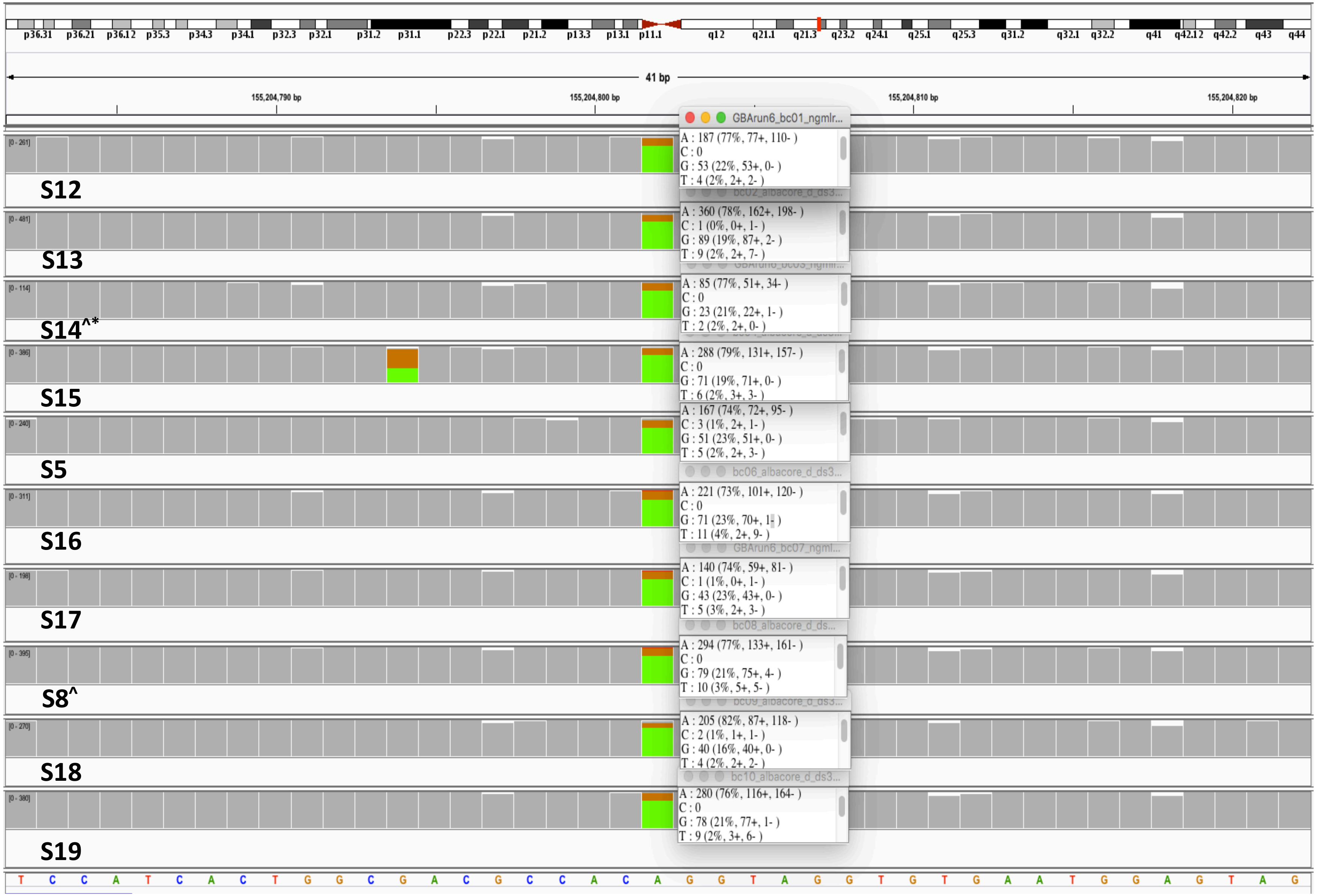

41 bp
155,204,790 bp
155,204,800 bp
155,204,810 bp
155,204,820 bp
GBArun6_bc01_ngmlr...
S12
A : 187 (77%, 77+, 110-)
C : 0
G : 53 (22%, 53+, 0-)
T : 4 (2%, 2+, 2-)
S13
A : 360 (78%, 162+, 198-)
C : 1 (0%, 0+, 1-)
G : 89 (19%, 87+, 2-)
T : 9 (2%, 2+, 7-)
S14^*
A : 85 (77%, 51+, 34-)
C : 0
G : 23 (21%, 22+, 1-)
T : 2 (2%, 2+, 0-)
S15
A : 288 (79%, 131+, 157-)
C : 0
G : 71 (19%, 71+, 0-)
T : 6 (2%, 3+, 3-)
S5
A : 167 (74%, 72+, 95-)
C : 3 (1%, 2+, 1-)
G : 51 (23%, 51+, 0-)
T : 5 (2%, 2+, 3-)
bc06_albacore_d_ds3...
S16
A : 221 (73%, 101+, 120-)
C : 0
G : 71 (23%, 70+, 1-)
T : 11 (4%, 2+, 9-)
GBArun6_bc07_ngml...
S17
A : 140 (74%, 59+, 81-)
C : 1 (1%, 0+, 1-)
G : 43 (23%, 43+, 0-)
T : 5 (3%, 2+, 3-)
bc08_albacore_d_ds...
S8^
A : 294 (77%, 133+, 161-)
C : 0
G : 79 (21%, 75+, 4-)
T : 10 (3%, 5+, 5-)
S18
A : 205 (82%, 87+, 118-)
C : 2 (1%, 1+, 1-)
G : 40 (16%, 40+, 0-)
T : 4 (2%, 2+, 2-)
bc10_albacore_d_ds3...
S19
A : 280 (76%, 116+, 164-)
C : 0
G : 78 (21%, 77+, 1-)
T : 9 (2%, 3+, 6-)
T C C A T C A C T G G C G A C G C C A C A G G T A G G G T G T G A A T G G A G T A G

(5)

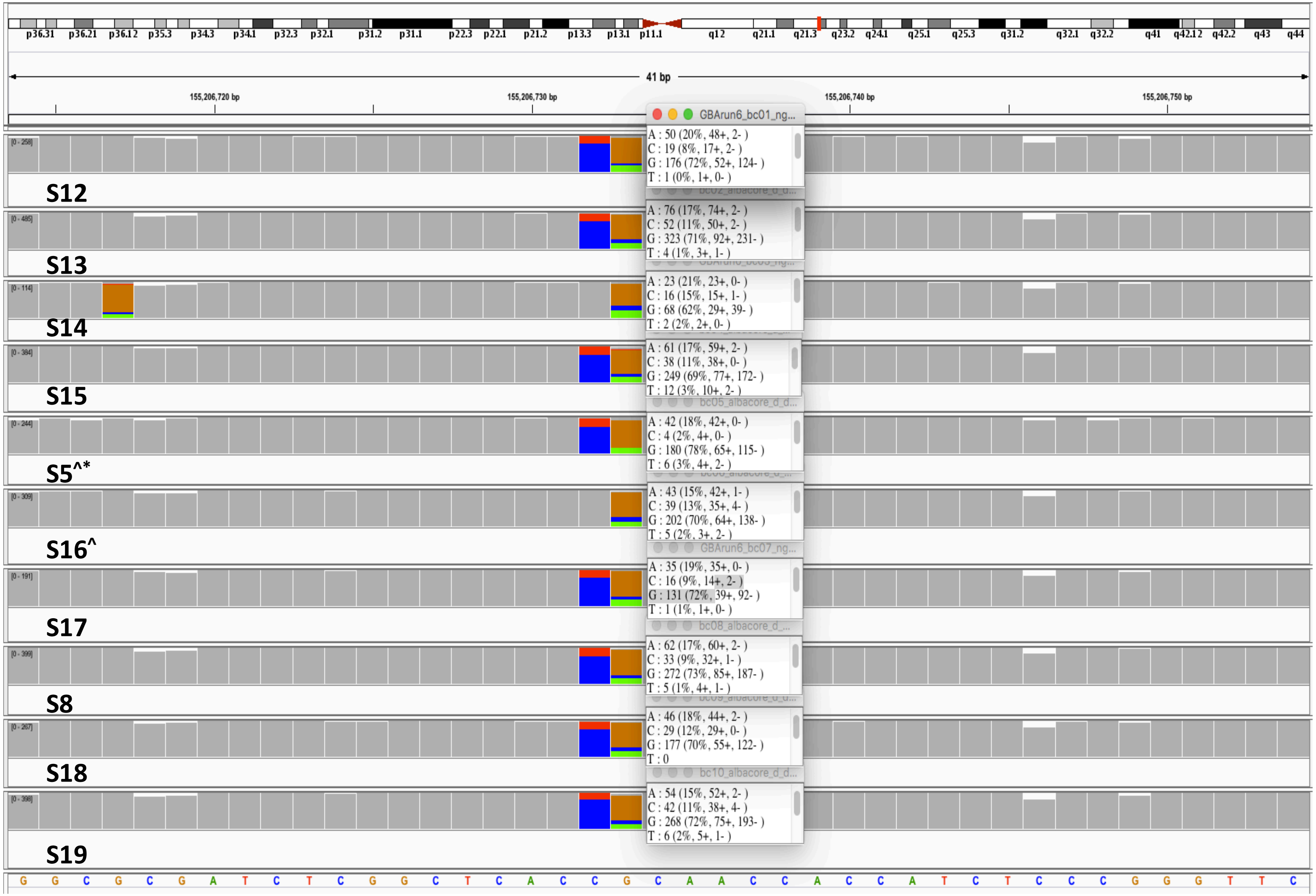

p36.31 p36.21 p36.12 p35.3 p34.3 p34.1 p32.3 p32.1 p31.2 p31.1 p22.3 p22.1 p21.2 p13.3 p13.1 p11.1 q12 q21.1 q21.3 q23.2 q24.1 q25.1 q25.3 q31.2 q32.1 q32.2 q41 q42.12 q42.2 q43 q44
41 bp
155,206,720 bp
155,206,730 bp
155,206,740 bp
155,206,750 bp
GBArun6_bc01_ng...
S12
A : 50 (20%, 48+, 2-)
C : 19 (8%, 17+, 2-)
G : 176 (72%, 52+, 124-)
T : 1 (0%, 1+, 0-)
S13
A : 76 (17%, 74+, 2-)
C : 52 (11%, 50+, 2-)
G : 323 (71%, 92+, 231-)
T : 4 (1%, 3+, 1-)
S14
A : 23 (21%, 23+, 0-)
C : 16 (15%, 15+, 1-)
G : 68 (62%, 29+, 39-)
T : 2 (2%, 2+, 0-)
S15
A : 61 (17%, 59+, 2-)
C : 38 (11%, 38+, 0-)
G : 249 (69%, 77+, 172-)
T : 12 (3%, 10+, 2-)
bc05_albacore_d_d...
S5^*
A : 42 (18%, 42+, 0-)
C : 4 (2%, 4+, 0-)
G : 180 (78%, 65+, 115-)
T : 6 (3%, 4+, 2-)
S16^
A : 43 (15%, 42+, 1-)
C : 39 (13%, 35+, 4-)
G : 202 (70%, 64+, 138-)
T : 5 (2%, 3+, 2-)
GBArun6_bc07_ng...
S17
A : 35 (19%, 35+, 0-)
C : 16 (9%, 14+, 2-)
G : 131 (72%, 39+, 92-)
T : 1 (1%, 1+, 0-)
bc08_albacore_d_...
S8
A : 62 (17%, 60+, 2-)
C : 33 (9%, 32+, 1-)
G : 272 (73%, 85+, 187-)
T : 5 (1%, 4+, 1-)
S18
A : 46 (18%, 44+, 2-)
C : 29 (12%, 29+, 0-)
G : 177 (70%, 55+, 122-)
T : 0
bc10_albacore_d_d...
S19
A : 54 (15%, 52+, 2-)
C : 42 (11%, 38+, 4-)
G : 268 (72%, 75+, 193-)
T : 6 (2%, 5+, 1-)
G G C G C G A T C T C G G C T C A C C G C A A C C A C C A T C T C C C G G G T T C

(6)

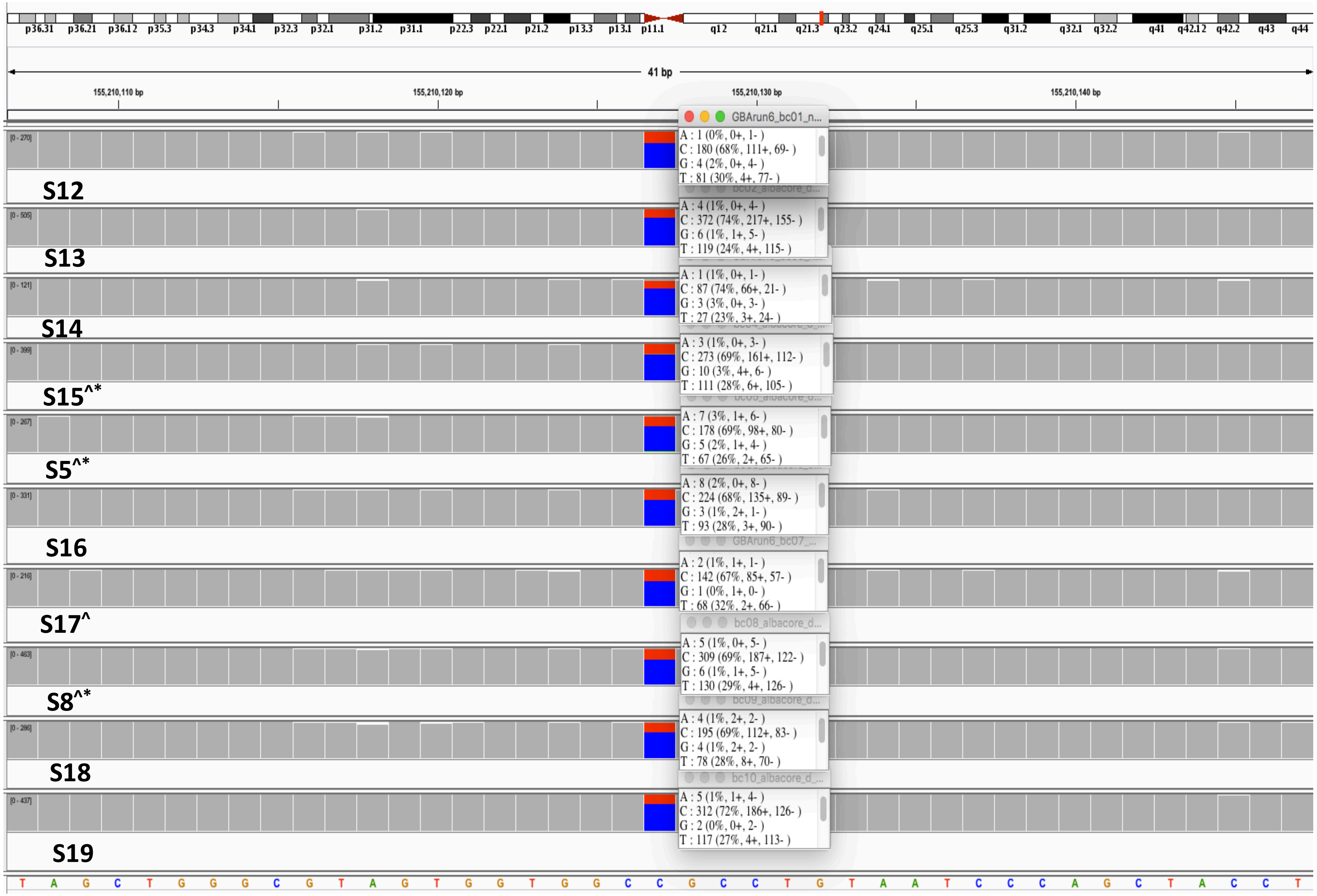
41 bp
155,210,110 bp
155,210,120 bp
155,210,130 bp
155,210,140 bp
GBArun6_bc01_n...
A : 1 (0%, 0+, 1-)
C : 180 (68%, 111+, 69-)
G : 4 (2%, 0+, 4-)
T : 81 (30%, 4+, 77-)
S12
A : 4 (1%, 0+, 4-)
C : 372 (74%, 217+, 155-)
G : 6 (1%, 1+, 5-)
T : 119 (24%, 4+, 115-)
S13
A : 1 (1%, 0+, 1-)
C : 87 (74%, 66+, 21-)
G : 3 (3%, 0+, 3-)
T : 27 (23%, 3+, 24-)
S14
A : 3 (1%, 0+, 3-)
C : 273 (69%, 161+, 112-)
G : 10 (3%, 4+, 6-)
T : 111 (28%, 6+, 105-)
S15^*
A : 7 (3%, 1+, 6-)
C : 178 (69%, 98+, 80-)
G : 5 (2%, 1+, 4-)
T : 67 (26%, 2+, 65-)
S5^*
A : 8 (2%, 0+, 8-)
C : 224 (68%, 135+, 89-)
G : 3 (1%, 2+, 1-)
T : 93 (28%, 3+, 90-)
S16
GBArun6_bc07_...
A : 2 (1%, 1+, 1-)
C : 142 (67%, 85+, 57-)
G : 1 (0%, 1+, 0-)
T : 68 (32%, 2+, 66-)
S17^
bc08_albacore_d...
A : 5 (1%, 0+, 5-)
C : 309 (69%, 187+, 122-)
G : 6 (1%, 1+, 5-)
T : 130 (29%, 4+, 126-)
S8^*
A : 4 (1%, 2+, 2-)
C : 195 (69%, 112+, 83-)
G : 4 (1%, 2+, 2-)
T : 78 (28%, 8+, 70-)
S18
bc10_albacore_d...
A : 5 (1%, 1+, 4-)
C : 312 (72%, 186+, 126-)
G : 2 (0%, 0+, 2-)
T : 117 (27%, 4+, 113-)
S19
T A G C T G G G C G T A G T G G T G G C C G C C T G T A A T C C C A G C T A C C T

(7)

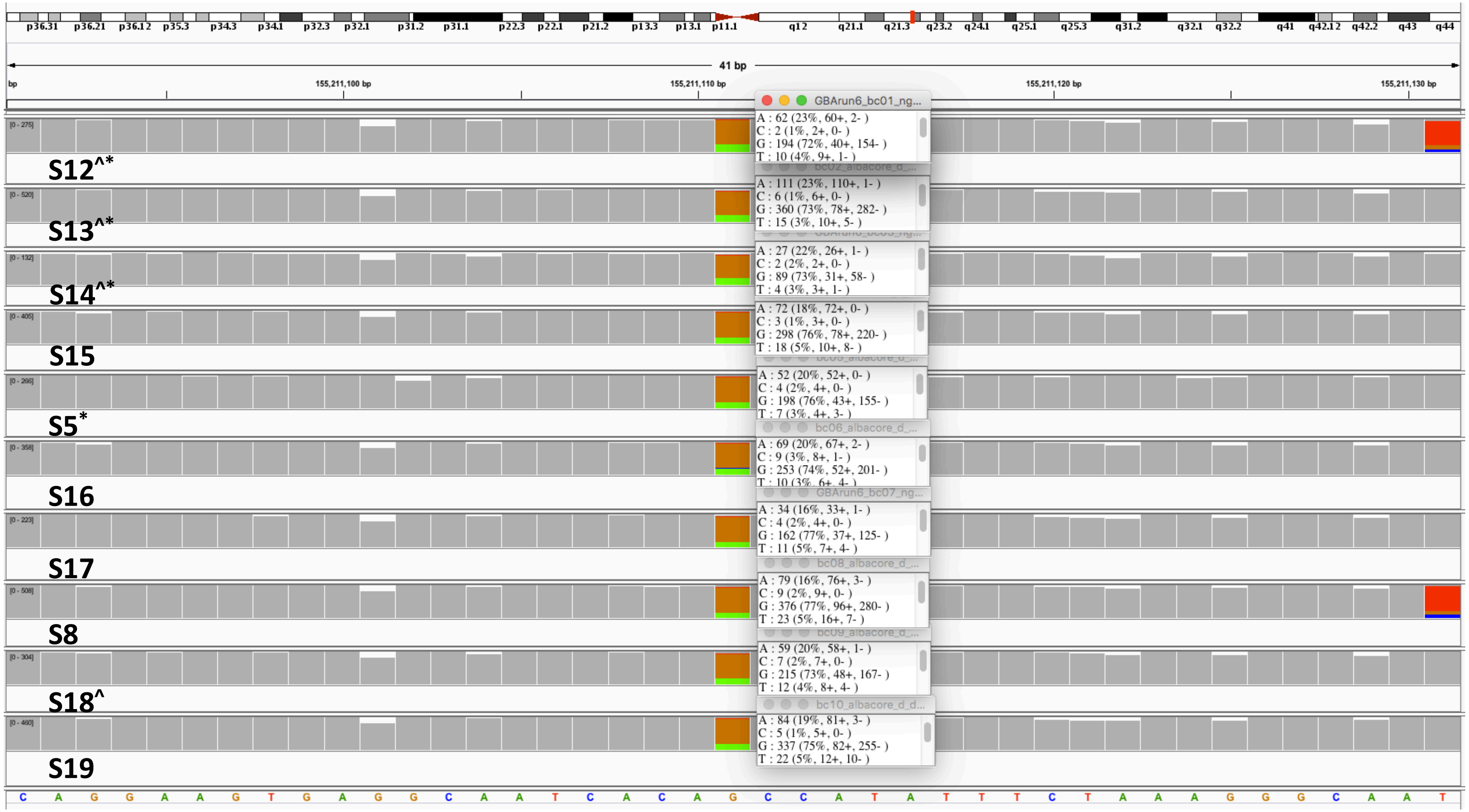
p36.31 p36.21 p36.12 p35.3 p34.3 p34.1 p32.3 p32.1 p31.2 p31.1 p22.3 p22.1 p21.2 p13.3 p13.1 p11.1 q12 q21.1 q21.3 q23.2 q24.1 q25.1 q25.3 q31.2 q32.1 q32.2 q41 q42.12 q42.2 q43 q44
41 bp
155,211,100 bp
155,211,110 bp
155,211,120 bp
155,211,130 bp
GBArun6_bc01_ng...
A : 62 (23%, 60+, 2-)
C : 2 (1%, 2+, 0-)
G : 194 (72%, 40+, 154-)
T : 10 (4%, 9+, 1-)
S12^*
A : 111 (23%, 110+, 1-)
C : 6 (1%, 6+, 0-)
G : 360 (73%, 78+, 282-)
T : 15 (3%, 10+, 5-)
S13^*
A : 27 (22%, 26+, 1-)
C : 2 (2%, 2+, 0-)
G : 89 (73%, 31+, 58-)
T : 4 (3%, 3+, 1-)
S14^*
A : 72 (18%, 72+, 0-)
C : 3 (1%, 3+, 0-)
G : 298 (76%, 78+, 220-)
T : 18 (5%, 10+, 8-)
S15
A : 52 (20%, 52+, 0-)
C : 4 (2%, 4+, 0-)
G : 198 (76%, 43+, 155-)
T : 7 (3%, 4+, 3-)
S5*
bc06_albacore_d_...
A : 69 (20%, 67+, 2-)
C : 9 (3%, 8+, 1-)
G : 253 (74%, 52+, 201-)
T : 10 (3%, 6+, 4-)
S16
GBArun6_bc07_ng...
A : 34 (16%, 33+, 1-)
C : 4 (2%, 4+, 0-)
G : 162 (77%, 37+, 125-)
T : 11 (5%, 7+, 4-)
S17
bc08_albacore_d_...
A : 79 (16%, 76+, 3-)
C : 9 (2%, 9+, 0-)
G : 376 (77%, 96+, 280-)
T : 23 (5%, 16+, 7-)
S8
A : 59 (20%, 58+, 1-)
C : 7 (2%, 7+, 0-)
G : 215 (73%, 48+, 167-)
T : 12 (4%, 8+, 4-)
S18^
bc10_albacore_d_d...
A : 84 (19%, 81+, 3-)
C : 5 (1%, 5+, 0-)
G : 337 (75%, 82+, 255-)
T : 22 (5%, 12+, 10-)
S19
C A G G A A G T G A G G C A A T C A C A G C C A T A T T T C T A A A G G G C A A T

Supplementary Figure S7. Downsampling and Nanopolish score review in three samples from first R9.4 flow cell.

A: After downsampling to ~50, 100, and 200 reads, the quality score was plotted against the total number of reads over that base for each. The highest number of reads is the original file (before downsampling). The mutation(s) carried in each sample are shown, with false positive calls (falsepos). Note that Nanopolish detected true variants regardless of reads. One false positive was found in all downsampled files, while others only at higher or lower coverage.

B: Two mutations in S15 (p.R502C and p.R535C) visualised on IGV showing uncorrected reads carrying each base, with and without downsampling.

C: Nanopolish adjusted quality score (absolute score divided by reads over that position) for true positive calls with and with downsampling as above.

A

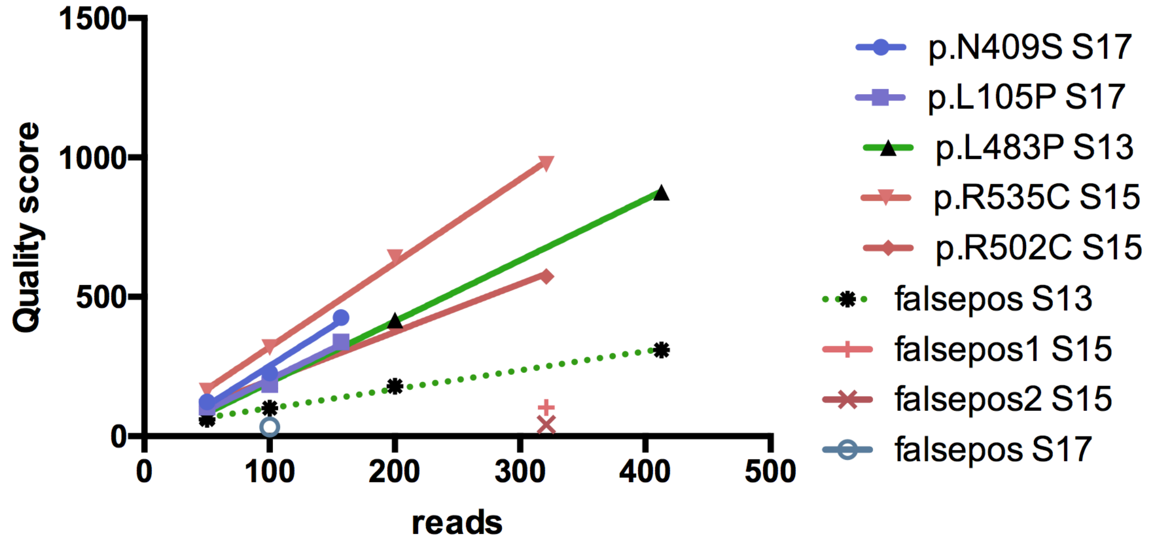

B

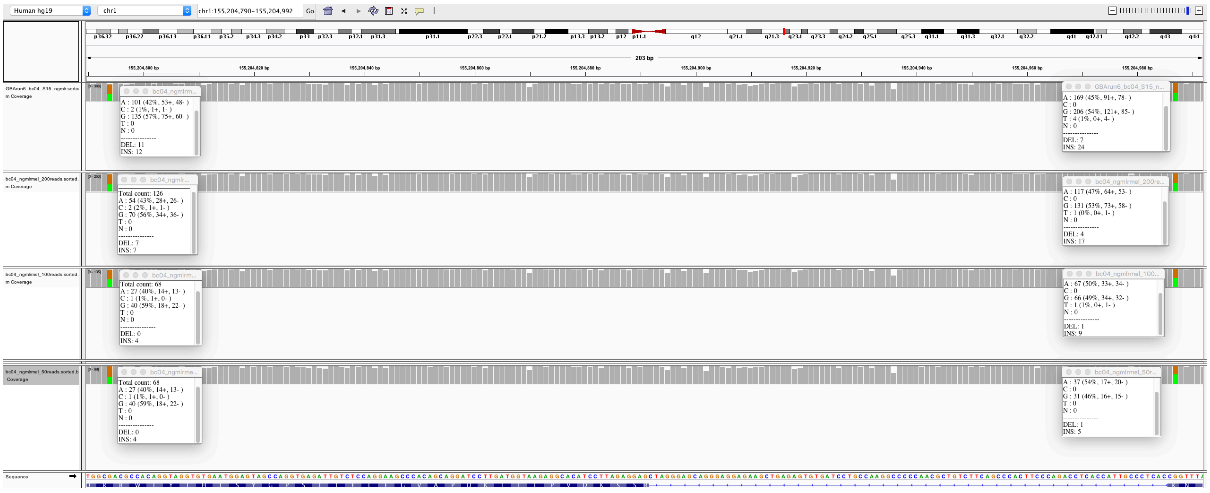

C

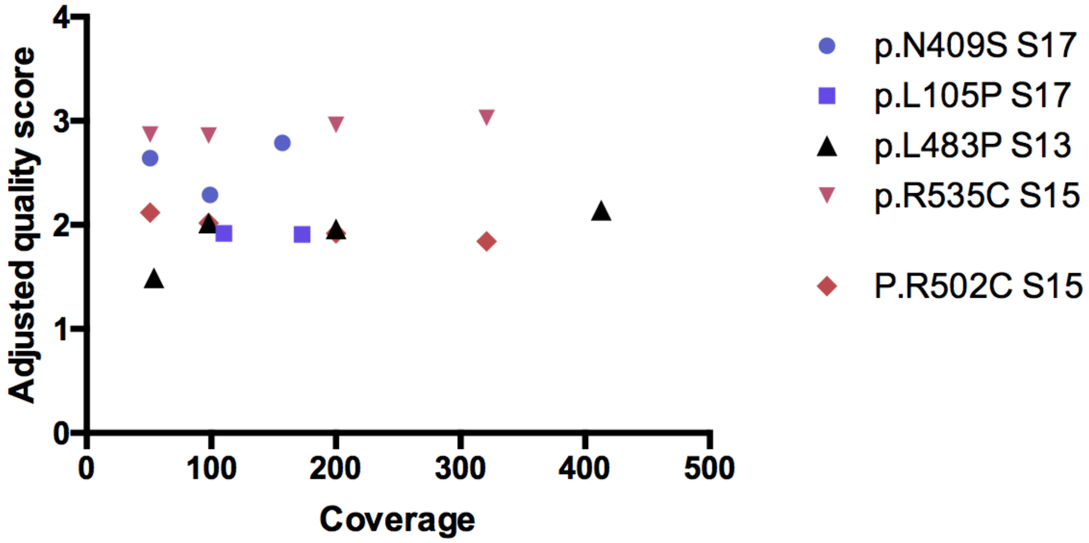
Adjusted quality score
Coverage
0
1
2
3
4
0
100
200
300
400
500
p.N409S S17
p.L105P S17
p.L483P S13
p.R535C S15
P.R502C S15

Supplementary Figure S8. Visualisation of 55-base pair exonic deletion in S5 with different aligners, and Sanger sequencing.

A: NGMLR alignment shows clear drop in coverage, and reads spanning deletion.

B: Graphmap alignment does not clearly reveal deletion.

C: Sanger sequencing shows two peaks per position from the start of deletion (sequenced in reverse direction).

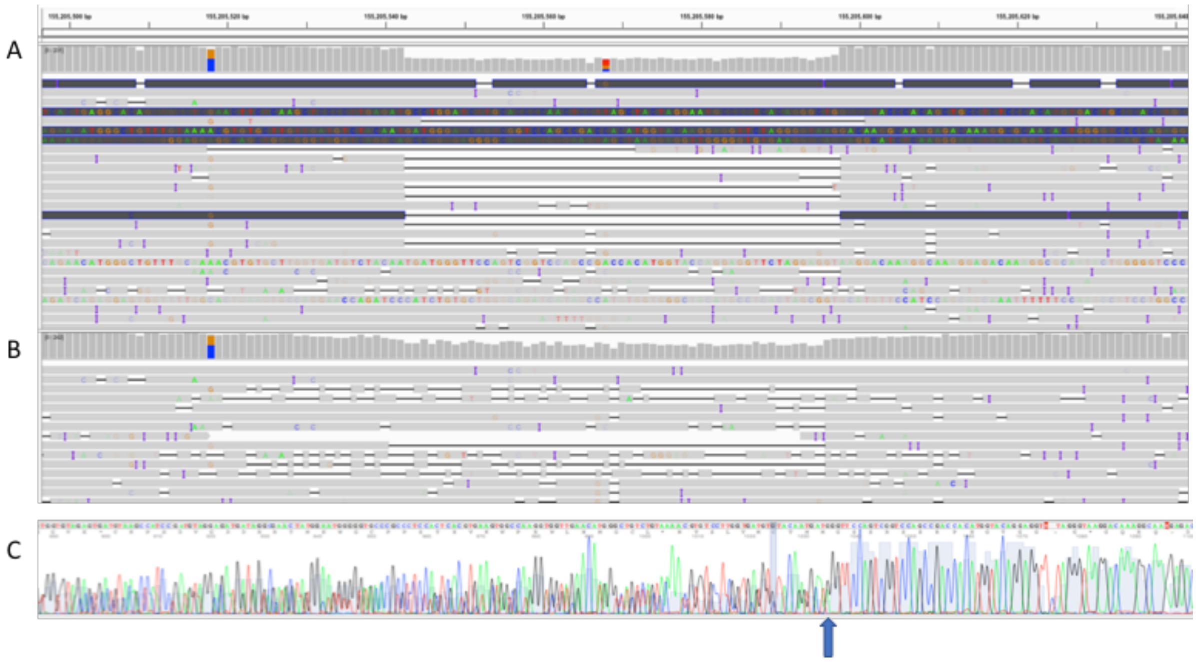

Supplementary Figure S9. Downsampling and Nanopolish score review in four samples from second R9.4 flow cell.

A: Quality score plotted against number of total over that position, including downsampling by factors of 2, 4, and 10. Note that the curve for a mutation present in different samples is almost identical across samples (three p.N409S, two p.L483P).

B: Nanopolish adjusted quality score (absolute score divided by reads over that position) for true positive calls with and with downsampling as above.

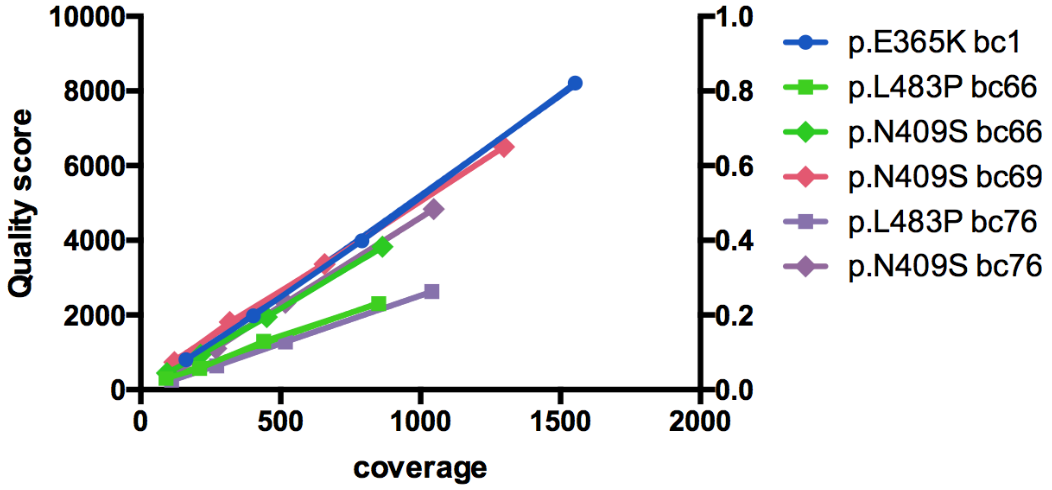

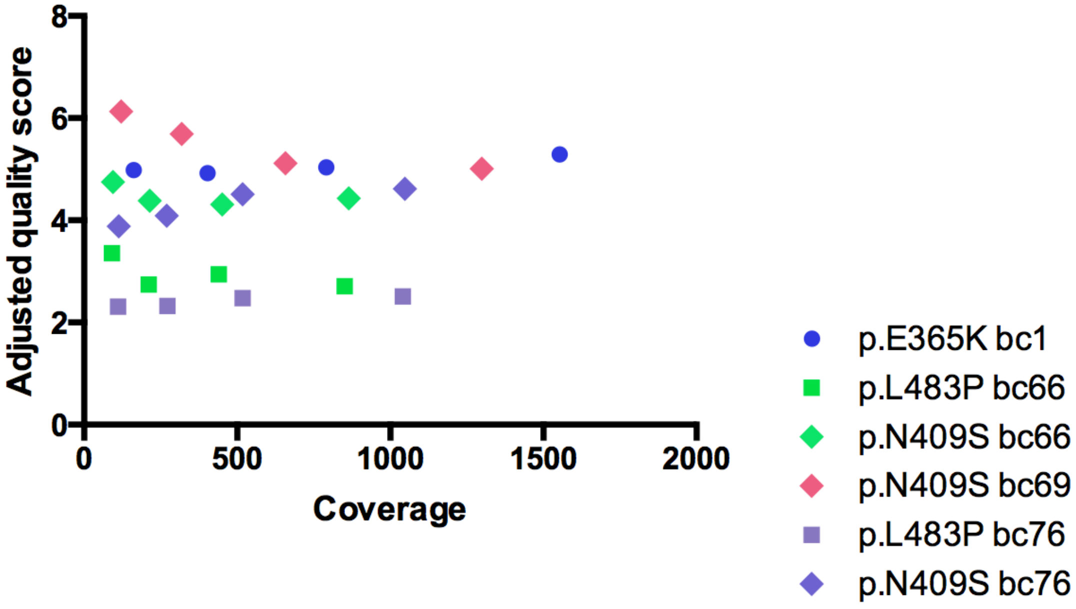

Supplementary Figure S10. Visualisation of GBA in the NA12878 MinION WGS publicly available data.

Top: all reads. Bottom: file filtered by samtools for mapping quality 1, to retain only reads with unique alignments. Note that these were aligned with BWA-MEM using the “-x ont2d” option.

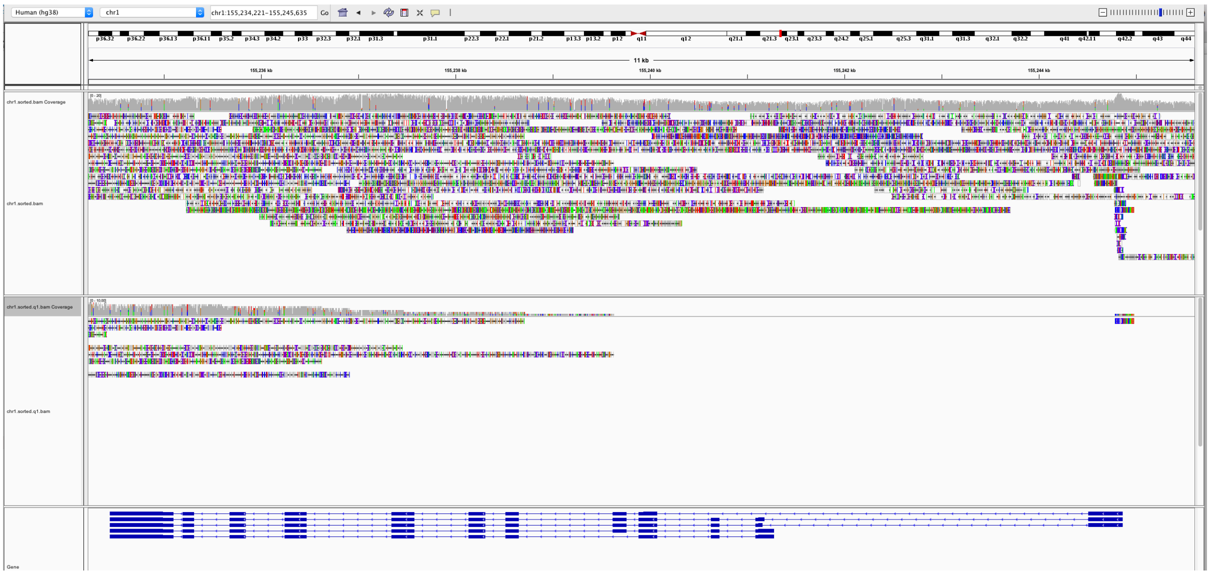

Supplementary Figure S11. Schematic explanation of possible recombinant variants.

These are simplified diagrams of possible but unverified local genomic structures. Details of recombination configurations and examples of fully resolved rearrangements have been reported (Spataro *et al.*, 2017; Tayebi *et al.*, 2003).

A: Normal configuration. *GBA* shown in blue, pseudogene in red. Intergenic regions coloured for clarity. The primer locations we used are shown as arrows. See also figure S1.

B: Likely structure of the recombination we detected, with pseudogene sequence inserted into gene, but primer binding sequences retained.

C: Possible structure of the recombinant we did not detect, as the primer sequence was deleted during the gene fusion event.

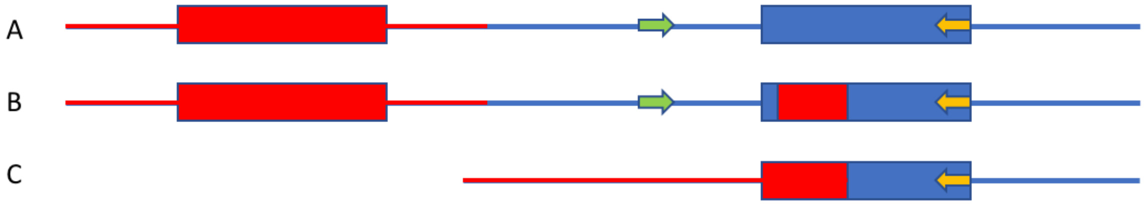

Supplement: Supplementary file 1 [file MGG3-7-na-s001.pdf]
