# Supplementary material for: Evaluation of the detection of GBA missense mutations and other variants using the Oxford Nanopore MinION
Source: Mol Genet Genomic Med. 2019 Jan 13;7(3):e564. doi: 10.1002/mgg3.564 (PMC6418358; doi:10.1002/mgg3.564)
Supplement: Supplementary file 2 [file MGG3-7-na-s002.pdf]

Supplementary Table S1. All samples with mutations found in this study.

Samples labelled "S" (DNA was from brain- substantia nigra) were analysed in the first 9.4 1D run (1D-1). S5 and S8 had also been analysed in the 2D early runs. Samples labelled "bc" were analysed in the second R9.4 1D run (1D-2). Zyg= zygosity, compl= compound heterozygote. Qual= Nanopolish quality score. Qual adjusted= score divided by total reads over that base in "polished" vcf.

Sample	Diagnosis	Mutation(s)	Zyg	Qual	Qual adjusted
S5	PD	c.1263del+RecTL	Het	(N/A)	
S8	PD	p.L483P	Het	630.3	2.0
S12	GD	p.N409S	Hom	1618.9	7.78
S13	GD relative	p.L483P	Het	875.5	2.2
S14	GD	p.N409S	Hom	702.1	7.89
S15	GD	p.R535C<br>p.R502C	Comp	975.5<br>572.7	3.19<br>1.87
S17	GD	p.N409S<br>p.L105P	Comp	425.5<br>337.8	2.84<br>2.00
S19	GD relative	p.L483P	Het	728.5	2.40
Bc01	PD	p.E365K	Het	7220.1	4.97
Bc05	PD	p.V433L	Het	985.4	4.90
Bc15	PD	p.T408M	Het	3975.9	3.59
Bc32	PD	p.T408M	Het	762.6	2.92
Bc47	PD	p.E365K	Het	8210.1	5.29
Bc51	Relative of PD	p.R209P	Het	788	4.78
Bc54	GD	p.N409S	Hom	9130.2	9.82
Bc56	GD	p.N409S<br>p.L483P	Comp	2857.2<br>1364.1	4.79<br>2.84
Bc58	GD	p.N409S	Hom	8624.2	9.58
Bc59	GD relative	p.N409S	Het	696.9	4.12
Bc60	GD	IVS9+1<br>p.N409S	Comp	1255.7<br>1118.9	5.13<br>4.44
Bc61	GD	p.L144R<br>p.N409S	Comp	515.7<br>551.9	4.00<br>4.38
Bc62	GD	p.N409S	Comp	282.1	2.82

		p.L483P		242.3	2.50
Bc63	GD relative	p.V433L	Het	7439.1	5.95
Bc65	GD	p.N409S<br>p.R398*	Comp	5261.8<br>5009.1	4.15<br>3.93
Bc66	GD	p.N409S<br>p.L483P	Comp	1769.6<br>1018.6	4.26<br>2.42
Bc67	GD relative	p.L483P	Het	1734.8	2.58
Bc68	GD relative	p.N409S	Het	1952.9	5.35
Bc69	GD relative	p.N409S	Het	1223.9	5.00
Bc71	GD relative	p.L483P	Het	2306.2	2.71
Bc73	GD relative	p.N409S	Het	1638.8	4.25
Bc74	GD	p.R301G<br>(RecNciI- missed)	Comp	6216.9	9.65
Bc76	GD	p.N409S<br>p.L483P	Comp	4834.3<br>2626.7	4.62<br>2.51
Bc79	GD relative	p.N409S	Het		
Bc80	GD relative	p.N409S	Het	6500.5	5.01
Bc82	GD	IVS9+1<br>p.N409S	Comp	2927.2<br>2779.2	5.01<br>4.73
Bc83	GD	p.V486E<br>p.N409S	Comp	628.2<br>829.1	3.4<br>4.58
Bc86	GD relative	p.N409S	Het	5054.5	4.14
Bc87	PD	p.N409S	Het	2877.1	4.26

Supplementary Table 2. PCR conditions for *GBA* amplification

Conditions		Protocol	
		Roche	Kapa
Reaction	DNA input (ng)	250-400 ng	100 ng
	Buffer	Buffer 2	2x HiFi HotStart ReadyMix
	Primers	npGBA-F & npcMTX1	npGBA-F & npcMTX1
	Enzyme	Expand Long template	HiFi HotStart
	Final reaction volume (µL)	50	50
Programme	Cycles	35	28
	Initial denaturation	94°C/2min	95°C/3min
	Denaturation	94°C/10sec	98°C/20sec
	Annealing	57°C/30sec	60.9°C/15sec
	Extension	68°C /7min (increasing 20 sec each cycle from 11 onwards)	72°C/9min
	Final extension	68°C/9min	72°C/9min

Supplementary Table S3. Summary of all runs. The sequencing conditions, read metrics, PCR protocol used, and mean aligned base identity assessment (ABID) by NanoOK are shown for each sequencing run. Barcoding was used in all runs expect the first one. Bam files from the same samples in runs 1-5 were merged prior to variant calling. (*) This flow cell was reused after washing, leading to very low yield. (**) Only 85 yielded coverage >100 over exons and were analysed. Review of failed samples showed smeary genomic DNA on agarose.

Run parameters	Run number						
	**2D-1**	**2D-2**	**2D-3**	**2D-4**	**2D-5**	**1D-1**	**1D-2**
**Type of reads**	2D					1D	1D
**Flow cell version**	R7.3	R7.3	R7.3*	R7.3	R9	R9.4	R9.4
**Run time (hours)**	2	48	46	48	48	48	48
**PCR protocol**	Roche	Roche	Roche	Mostly Kapa	Mostly Kapa	Kapa	Kapa
**Samples included**	1	9	3	7	10	10	92**
**Mean ABID (%) ± SD**	94.62	94.96 ±0.19	94.84 ±0.29	94.78 ±0.76	96.45 ±1.55	93.56 ±1.21	

Supplementary table S4. Detailed data for 2D runs 1-5.

Samples tested, PCR protocol used, NanoOK metrics, alignment comparison, and total numbers of SNPs called. NanoOK quality assessment after alignment to the target sequence (chr1:155202292-155211206) is shown. The coverage of the gene and pseudogene (equivalent region over MTXN-GBAP chr1:155181579-155189601) after Graphmap alignment to the whole genome and merging bam files from different runs is also given. Total SNPs includes the coding ones in S5 and S8. Note that S7 was included in runs 4 and 5 as separately barcoded samples, amplified with different PCR enzymes. There was a possible minimal improvement in ABID with Kapa in both cases (1% and 0.1%). Two additional brain samples, coded S9 and S10, only gave a few correctly aligned reads, and were not analysed further.

Sample	Experiment		NanoOK analysis				Coverage		Total non-coding SNP
	Run ID	PCR protocol	N50	Reads aligned (%)	ABID (%)	IBAB (%)	Gene	Pseudogene	
**S1**	1	Roche	8783	94.6	95.0	85.5	579	11	12
	2	Roche	8734	37.5	95.4	85.8			
	3	Roche	8775	40.4	95.4	85.9			
	4	Kapa	8464	100.0	95.7	85.4			
	5	Kapa	8454	33.1	97.4	89.9			
**S2**	2	Roche	8737	44.0	94.8	84.5	63	0	2
**S3**	2	Roche	8714	48.6	95.0	85.0	359	6	10
	3	Roche	8752	46.0	94.8	84.7			
	4	Kapa	8685	86.2	95.7	86.2			
	5	Kapa	8486	74.9	97.3	89.8			
**S4**	2	Roche	8687	33.1	95.1	84.9	79	1	2
	5	Kapa	524	4.9	93.5	82.9			
**S5**	2	Roche	8725	36.1	95.0	84.6	235	2	8
	3	Roche	8824	41.7	94.9	84.8			
	4	Roche	8632	87.5	95.3	85.5			
	5	Roche	8663	86.5	97.4	90.1			
**S6**	2	Roche	8740	39.4	89.5	84.7	113	2	7
	5	Kapa	4215	8.9	98.1	91.6			
**S7**	2	Roche	8722	31.2	94.7	84.3	291	0	12
	4	Roche/Kapa	8595/8603	100/100	94.7/95.7	84.7/85.9			
	5	Roche/Kapa	8660/8572	87.9/74.1	97.3/97.4	89.9/90.1			
**S8**	2	Roche	8726	64.7	95.1	85.0	1095	9	2
	4	Kapa	8649	98.2	95.7	86.0			
	5	Kapa	8618	88.8	97.5	90.2			
**S11**	2	Roche	8743	38.6	94.9	84.7	70	1	6
	5	Kapa	761	4.9	96.1	88.1			
						**Mean**	**320.4**	**3.6**	**6.8**

Supplementary Table S5. Run 1D-1: NanoOK metrics, and coverage of gene and pseudogene (GBAP) by each aligner.

Sample	NanoOK metrics				Coverage by each aligner			
					Graphmap		NGMLR	
	N50	% reads aligned	ABID (%)	IBAB (%)	GBA	GBAP	GBA	GBAP
S5	8851	73.5	94.6	84.3	250.7	3.4	234.8	11.7
S8	4052	32.3	92.5	81.3	435.2	4.8	427.2	9.0
S12	8440	40.5	94.6	84.5	270.5	2.2	264.0	4.3
S13	8737	55.3	94.5	84.3	499.1	4.6	496.6	7.1
S14	3976	31.3	92.4	81.3	121.8	3.2	119.1	2.9
S15	7465	45.3	93.6	82.9	400.3	1.7	392.2	7.0
S16	3634	27.5	91.4	79.9	331.9	0.7	322.7	5.6
S17	4543	38.0	93.6	82.9	208.0	2.2	205.1	3.3
S18	8888	64.6	94.8	84.9	285.6	0.5	280.2	3.4
S19	3865	24	92.0	79.7	415.9	1.2	409.3	7.4
**Mean**					322	2.5	315	6.2
**% of coverage of GBAP to gene**					0.78		1.97	

Supplementary Table S6. Intronic SNPs reported in run 1D-1.

The position and ID for each SNP is given. For each SNP called in a sample, the Nanopolish quality score for NGMLR alignment is given: the absolute number, and the score adjusted by number of reads in brackets (see text).

		Sample	S5	S8	S12	S13	S14	S15	S16	S17	S18	S19
	Position and ID	Base change										
1	155202689 rs10796940	A>C				1161.7 (2.9)		2307.4 (7.6)				881.7 (2.8)
2	155203060 rs11264344	G>A				754 (1.9)		1569.6 (5.3)				606.2 (2.0)
3	155204694 rs708606	C>T	546.9 (3.2)									
4	155205170 rs2974924	A>G	533 (3.1)									
5	155205203 rs426516	G>A	551.4 (3.2)									
6	155205331 rs1800473	T>C	677.1 (3.7)									
7	155205657 rs111417507	A>G	1458.5 (7.7)									
8	155205669 rs3115534	G>T	1458.5 (7.7)									
9	155205709 rs548435731	A>G	822.2									

			(4.3)									
10	155206341<br>rs9628662	T>G				1478.9<br>(3.6)		2664.4<br>(8.7)				1433.2<br>(4.5)
11	155207030<br>rs72704130	G>A						1211<br>(3.9)				
12	155207549<br>rs762488	T>C				721.6<br>(1.8)		1486.9<br>(4.7)				
13	155207626<br>rs2009578	G>A				1392.2<br>(3.4)		2624.3<br>(8.4)				684.2<br>(4.0)
13	155208183<br>rs2974923	T>C				1011.9<br>(2.5)		1745.2<br>(5.6)				834.7<br>(2.6)
14	155208647<br>rs7416991	T>C	1353.3<br>(7.1)	2303.3<br>(6.9)	1479.3<br>(7.3)	2727<br>(6.6)	588.2<br>(6.5)		1776.7<br>(6.9)	1079.2<br>(6.8)		2228.6<br>(6.7)
15		T>G						2268.3<br>(7.4)				
16	155209360<br>rs2075569	C>T				916.5<br>(2.2)		1614.3<br>(5.0)				707.2<br>(2.0)

Supplementary Table S7. False positives reported in run 1D-1.

False positive number 3 was reported in all Graphmap-aligned samples, but not in NGMLR. For each SNP called in a sample, the Nanopolish quality score for NGMLR alignment is given: the absolute number, and the score adjusted by number of reads in brackets (see text). The total false positive calls per sample with each aligner is shown at the end.

		Sample	S5	S8	S12	S13	S14	S15	S16	S17	S18	S19
1	155203446	G>A					105.3 (1.2)					
2	155203545	C>T						103.4 (0.3)				
3	155204190	G>A										
4	155204802	A>G	99.4 (0.6)						127.1 (0.5)			
5	155206733	G>A					24.6 (0.3)					
6	155210127	C>T	29.2 (0.1)	26.8 (0.2)				41.3 (0.1)				
7	155211111	G>A	143.6 (0.6)		175.2 (0.8)	309.3 (0.7)	113.1 (1.0)					
False positive calls by aligner		Graphmap	3	3	2	2	4	2	1	2	2	1
		NGMLR	3	1	1	1	3	2	1	0	0	0

Supplementary table S9. False positives reported in run 1D-2

The positions, change, average adjusted nanopolish score quality, and number of samples in which it was reported are shown**.** Note that the two with the highest scores had been found before, and the scores of the new ones were very low.

POS	REF	ALT	QUAL adj	samples
155204325	C	T	0.18	80
155204802	A	G	1.16	4
155204986	C	T	0.11	5
155205458	C	T	0.15	1
155208056	G	A	0.07	14
155208082	G	A	0.11	48
155211111	G	A	1.57	4
